# Integrated meta-omics reveals the regulatory landscape involved in lipid metabolism between pig breeds

**DOI:** 10.1186/s40168-023-01743-3

**Published:** 2024-02-20

**Authors:** Jiajie Sun, Fang Xie, Jing Wang, Junyi Luo, Ting Chen, Qingyan Jiang, Qianyun Xi, George E. Liu, Yongliang Zhang

**Affiliations:** 1 Guangdong Provincial Key Laboratory of Animal Nutrition Control, National Engineering Research Center for Breeding Swine Industry, College of Animal Science, South China Agricultural University, Guangzhou, Guangdong 510642 China; 2 Animal Genomics and Improvement Laboratory, USDA-ARS, BARC-East, Beltsville, MD 20705 USA; 3 Institute of Animal Husbandry and Veterinary Medicine, Henan Academy of Agricultural Sciences, Zhengzhou, 450002 China

**Keywords:** Animal model, Intermuscular fat, Single-cell RNA sequencing, Lipidomics, Metagenome

## Abstract

**Background:**

Domesticated pigs serve as an ideal animal model for biomedical research and also provide the majority of meat for human consumption in China. Porcine intramuscular fat content associates with human health and diseases and is essential in pork quality. The molecular mechanisms controlling lipid metabolism and intramuscular fat accretion across tissues in pigs, and how these changes in response to pig breeds, remain largely unknown.

**Results:**

We surveyed the tissue-resident cell types of the porcine jejunum, colon, liver, and longissimus dorsi muscle between Lantang and Landrace breeds by single-cell RNA sequencing. Combining lipidomics and metagenomics approaches, we also characterized gene signatures and determined key discriminating markers of lipid digestibility, absorption, conversion, and deposition across tissues in two pig breeds. In Landrace, lean-meat swine mainly exhibited breed-specific advantages in lipid absorption and oxidation for energy supply in small and large intestinal epitheliums, nascent high-density lipoprotein synthesis for reverse cholesterol transport in enterocytes and hepatocytes, bile acid formation, and secretion for fat emulsification in hepatocytes, as well as intestinal-microbiota gene expression involved in lipid accumulation product. In Lantang, obese-meat swine showed a higher synthesis capacity of chylomicrons responsible for high serum triacylglycerol levels in small intestinal epitheliums, the predominant characteristics of lipid absorption in muscle tissue, and greater intramuscular adipcytogenesis potentials from muscular fibro-adipogenic progenitor subpopulation.

**Conclusions:**

The findings enhanced our understanding of the cellular biology of lipid metabolism and opened new avenues to improve animal production and human diseases.

Video Abstract

**Supplementary Information:**

The online version contains supplementary material available at 10.1186/s40168-023-01743-3.

## Introduction

Pork is the most consumed meat in China [1], though only slightly less than poultry meat globally in recent years [2]. The consumer acceptance and perception of pork are affected by numerous quality traits of meat [3]. In farm animals, the quantity and distribution of intramuscular fat (IMF), referred to as marbling fat, is highly desirable for enhancing meat quality [4], including tenderness, flavor, juiciness, firmness, and palatability [5]. In humans, muscle tissues accumulate IMF through age, diet, gender, and genetics [6], and excess accumulation of IMF is also responsible for metabolic abnormalities and is associated with a variety of increasing prevalence of obesity, insulin resistance, muscular dystrophies, and sarcopenia [6, 7]. In general, domestic pigs share genetic, anatomical, and physiological similarities with humans [8, 9] and therefore are considered to be an excellent model for studying lipid metabolic disease [10].

The adipose tissue is a specialized loose connective tissue and highly active metabolic endocrine organ that is composed mostly of adipocytes [11, 12]. In general, adipose cells are derived from mesenchymal stem cells (MSCs), further developing into a pool of progenitor cells with dual potentials of adipogenic and fibrogenic differentiation, thus are termed fibro-adipogenic progenitors (FAPs) [13]. FAPs commit into adipocyte lineage and eventually give rise to preadipocytes and then undergo terminal differentiation into mature adipocytes [14]. In animals, adipogenesis is initiated around midgestation with the formation of preadipocytes, the majority of which differentiate into mature adipocytes in late gestation or early postpartum period [15]; new adipocytes generated in adulthood are mainly located in subcutaneous, visceral, and retroperitoneal connective tissues, with few located in the intramuscular fat depot [16]. Thus, maternal nutritional management during pregnancy and lactation, which enhances the number of mesenchymal cells committed to intramuscular adipogenesis, will provide more sites for fat accumulation during fattening and further marbling [17]. This physiological process involves a complex homeostatic orchestration of diverse metabolic tissues, such as digestion and absorption of nutrients by the intestinal tract [18], as well as gut microbiota [19], metabolism and biotransformation with the liver [20], and communication with muscle tissue [21]. During the past decades, substantial progress has been made in identifying the dynamic and integrative functions of these key tissues in adipogenesis. However, the underlying cell types and composition of these tissues, and especially the functions of these cell subpopulations, are not completely understood.

In particular, the application of high-dimensional approaches, such as single-cell RNA sequencing (scRNA-seq), has revealed finer levels of heterogeneity across various tissues, providing in-depth snapshots of individual cell activity and function in several physiological and pathological processes [22]. A recent scRNA-seq study of chicken skeletal muscles elucidated the cell subtypes and captured the gene expression of individual cells, revealing novel insights into myogenesis and intramuscular adipogenesis [23]. Here, we performed scRNA-seq to construct the single-cell atlases of jejunum, colon, liver, and longissimus dorsi muscle between fat-type (Lantang, LT) and lean-type (Landrace, LW) piglets and revealed hitherto unappreciated cell-specific features across different tissues in lipid metabolism. Our study opens potential avenues to further improve pork quality for human consumption and broadens our understanding of the mechanisms that underpin lipid metabolic diseases.

## Results and discussion

**Fig. 1:**
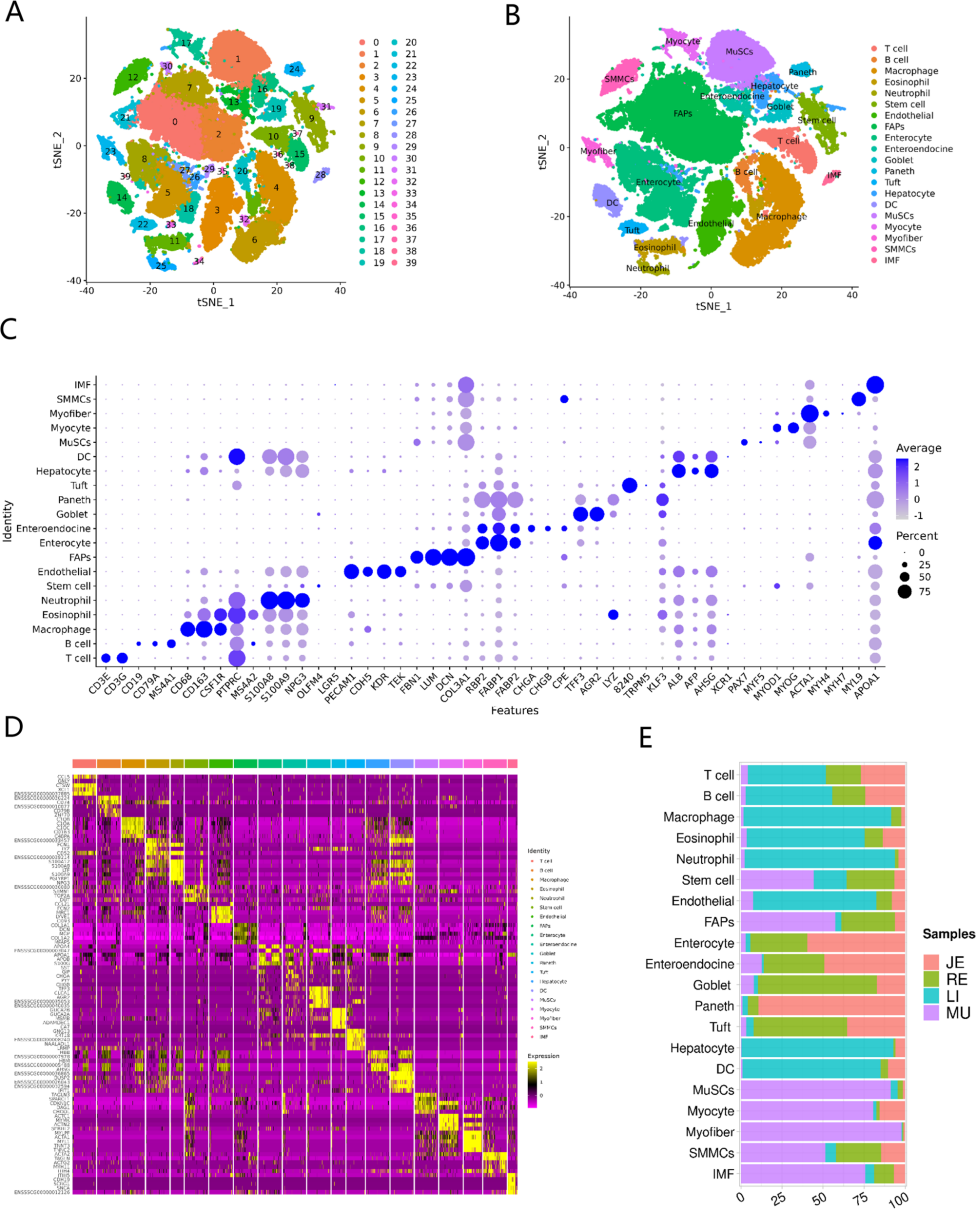
Construction of the single-cell landscape of porcine jejunum, colon, liver and muscle tissues. **A** tSNE plots showing the distribution of the main tissue-resident clusters in the tested tissues. **B** t-SNE analysis of 60,514 single cells from tested samples, with 20 major cell types labeled in different colors. IMF, intramuscular fat; SMMCs, smooth muscle cells; MuSCs, Muscle stem cells; DC, dendritic cells; FAP, fibroadipogenic precursors/myofibroblast. **C** Dot plots showing the expression of representative marker genes; dot color reflects average gene expression, and dot size represents percent of cells expressing the gene. **D** Heatmap representing the top 5 most variably expressed genes (row) between the given cell subpopulations (column); colors and numbers correspond to the cell types shown in B. **E** Proportions of the 20 major cell types in the tested tissues; JE, jejunum; RE, colon; LI, liver; MU, longissimus dorsi muscle

The domestic pig is an indispensable agricultural species for meat sources [1] and a powerful biomedical model for studying human developmental processes and diseases [24]. In general, intramuscular fat, described as marbling for meat quality, is defined as a unique adipose depot interspersed between and around myofiber groups, and in parallel, strongly associated with human health and disease [25]. Therefore, we systematically assessed cell type heterogeneity and transcriptional profiles of multiple tissues that are involved in lipid digestion, absorption, transport, conversion, and deposition between different breeds of pigs, which allowed us to propose a unified annotation of adipocytogenesis and lipogenesis processes at cellular levels in piglets. We profiled single-cell transcriptomes of the porcine jejunum, colon, liver, and longissimus dorsi muscle, with two for each tissue as biological replicates and one replicate for each breed, on a 10× genomics system. In total, we obtained more than 3405.28 million sequencing reads, and on average, more than 425.66 ± 17.17 million sequencing reads for each sample. After quality filtering, we retained a total of 60,514 high-quality single cells for downstream analysis, of which 10,227, 12,906, 18,152, and 19,229 belonged to the jejunum, colon, liver, and muscle, respectively. We then identified a total of 40 cell clusters using t-distributed stochastic neighbor embedding (t-SNE) analysis, with a resolution value = 0.8 (Fig. 1A). These clusters were divided into 20 major cell populations (Fig. 1B) based on the expression levels of canonical cell-type-specific markers (Fig. 1C; Table S1A) and the annotated functions of differentially expressed genes (DEGs) in each cluster (Fig. 1D; Table S1B). Consistent with the characteristics and function of various tissues, immune-related cells, such as T cells, B cells, macrophages, eosinophils, and neutrophils, clearly exhibited in liver tissue, as well as hepatocytes and dendritic cells; enterocytes, enteroendocrine cells, goblet cells, Paneth cells, and tuft cells, a series of intestinal epithelium cells, were significantly enriched in the jejunum and colon tissues, while multiple myogenesis-related subtypes and intramuscular adipocytes were mainly identified in the longissimus dorsi tissue (Fig. 1E). In recent, another study used a similar strategy to elucidate the chicken intermuscular fat cells in skeletal muscle [23], and in addition, our findings here fully corroborated this approach while extending it across additional tissues and breeds. To our knowledge, this study is one of the first to constitute a comprehensive resource describing the molecular signatures of porcine lipid metabolism across tissues, potentially benefiting the scientific community to better apprehend the lipogenesis and adipogenesis processes in humans.

### Characterization and function of porcine intestinal cells

**Fig. 2:**
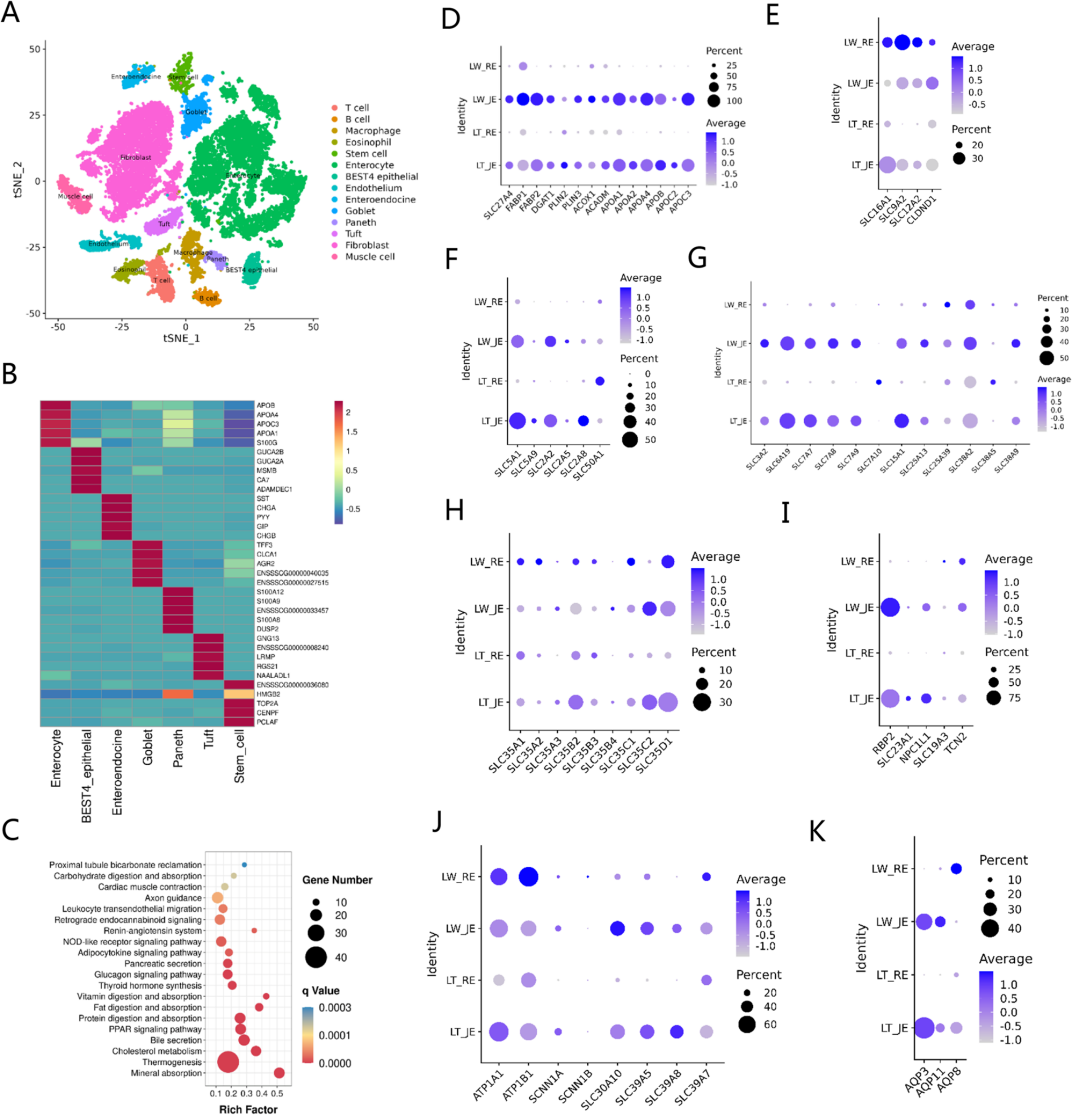
Cell landscapes and differential nutrient absorption preferences of porcine intestines based on single-cell transcriptome profiles. **A** The Seurat algorithm and t-SNE plot were used to visualize the cell subtypes of all 23,133 intestinal cells. **B** Expression heatmaps of the top 5 cell type-specific genes in various intestinal epithelial cells. **C** KEGG analysis of porcine enterocyte-enriched genes at the organismal systems level. **D**–**K** Expression patterns of specific genes involved in nutrient absorption and transport in different intestine segments, mainly including lipids (**D**), short-chain fatty acid (**E**), sugar (**F**), amino acids (**G**), nucleotide or nucleotide sugar (**H**), vitamin (**I**), metal ion (**J**), and water (**K**). Each dot represented a gene, of which the color saturation indicated the average expression level in an intestine segment, and the size indicated the percentage of cells expressing the gene

To characterize the cellular diversity of porcine intestines, a total of 23,133 intestinal cells were pooled together, and their transcriptome profiles were classified into 28 cell clusters with unsupervised graph-based clustering (Table S2A). We assigned cell identities based on a panel of previously established markers (Table S2B) and visualized the data using t-SNE. From this analysis, we identified 14 specific intestine-resident subtypes (Fig. 2A), including T cells, B cells, macrophages, eosinophils, stem cells, enterocytes, BEST4/OTOP2 epithelial cells, endothelium cells, enteroendocrine cells, goblet cells, Paneth cells, tuft cells, fibroblasts, and muscle cells. These major cell types were also identified in the jejunum and colon segments when analyzed separately (Fig. S1A-B). In total, we identified 16 and 14 specific jejunum- and colon-resident subtypes, including the major subpopulations identified in the mouse and human intestinal epithelium [26, 27]. Specifically, the previously described population of type 3 innate lymphoid cells (ILC3) was only identified in the small intestine with the expression of the *PTPRC*, *ETS1*, *TOX*, *IL7R*, *ID2*, and *RORC* genes (Fig. S1C), as well as pericytes stably expressing *KCNJ8*, *ABCC9*, and *RGS5* (Fig. S1D). In contrast, a small proportion of subpopulations were observed and termed as distal mature enterocytes in the large intestinal enterocytes with high expression of *EPCAM*, *FABP1*, *FAM3D*, and *AQP8* (Fig. S1E). Our data captured 12,409 epithelial cells, accounting for nearly half of high-quality intestinal cells (Table S2C), which were demarcated by highly specific gene expressions such as *EPCAM* and *FABP1* (Fig. S1F) and were readily distinguished on their top 5 gene signatures (Fig. 2B). In addition, t-SNE analysis of the enterocyte cluster revealed the presence of two juxtaposed clusters (Fig. S1G), and we observed substantial locational divergence between proximal (jejunal) and distal (colonic) population demarcated by specific gene expression of *FABP2*, *APOC3*, *AQP8*, and *SLC26A2* (Fig. S1H-I), in agreement with the previous description [26].

In general, the intestinal epithelium is the major organ for a wide range of nutrients digestion, absorption, and processing, including lipids, glucose, amino acids, vitamins, and metallic trace elements. In this study, we captured a total of 1321 significantly upregulated genes in the enterocyte subtype cells (Table S2D), and these candidates were enriched in a number of distinct categories at the organismal systems level based on KEGG analysis (Table S2E), such as mineral absorption, protein digestion and absorption, fat digestion and absorption, and carbohydrate digestion and absorption (Fig. 2C). In detail, the genes participating in lipid transport (*SLC27A4*, *FABP1*, *FABP2*), triglyceride assembly (*DGAT1*) [28], lipid homeostasis (*PLIN2* and *PLIN3*) [29], β-oxidation (*ACOX1* and *ACADM*), and lipid assimilation (*APOA1*, *APOA2*, *APOA4*, *APOB*, *APOC2*, and *APOC3*) [30] were highly expressed in the jejunum (Fig. 2D). In contrast, the genes related to short-chain fatty acid (SCFA) transport were enriched in the colon segment and obviously enriched in LW breed (Fig. 2E), including *SLC16A1*, *SLC9A2*, *SLC12A2*, and *CLDND1* [31]. High expression of the genes related to the transport of lipids in the jejunum indicated that lipid absorption was mainly accomplished in the small intestine, which was consistent with an earlier report [30], while the expression of the genes involved in the transport of SCFA was obviously enriched in the colon segments, which were also in line with previous findings [32]. Both the jejunum and colon were involved in sugar absorption (Fig. 2F), but the small intestine specially transported monosaccharides such as glucose, fructose, galactose, and xylose based on the enriched expression of *SLC5A1*, *SLC2A2*, and *SLC2A8* [33], while the large intestine transported aldoses, including pentoses and hexoses, via the expression of *SLC50A1* [34]. The transporters expressed for amino acids in the intestinal segments are mainly members of the *SLC3A*, *SLC6A*, *SLC7A*, *SLC15A*, *SLC25A*, and *SLC38A* families (Fig. 2G) [30–33]. The channels of SLC3A2, SLC25A13, and SLC25A39 for essential amino acid transport were mainly expressed in both the jejunum and colon, especially in the jejunum [35], while SLC6A19, SLC7A7, SLC7A8, and SLC7A9 were enriched in jejunum for neutral and cationic amino acid transport such as leucine or arginine [33]. In addition, *SLC38A9* is specifically expressed in the jejunum and exhibits low amino acid-sensing activity for arginine transport [36]. *SLC15A1* was substantially expressed in the jejunum tissue and responsible for the absorption of different di- and tripeptides [37, 38]. In the colon segment, *SLC7A10* and *SLC38A5* were highly expressed and mainly moved small neutral amino acids such as alanine, serine, cysteine, glycine, and threonine [39], while glutamine transporter SLC38A2 displayed similar expression patterns in the jejunum and colon tissues [40]. Our data also suggested that the colon was the major site for nucleotide or nucleotide sugar absorption with the SLC35 family (Fig. 2H) [41], in agreement with an earlier report [30]. For vitamin (Fig. 2I), the genes related to vitamin A, C, D, and E absorption were enriched in the jejunum, such as *RBP2* for the absorption of vitamin A [42], *SLC23A1* for vitamin C [43], and *NPC1L1* for vitamin D and E [44, 45], while the whole intestinal segments could transport vitamin B1, B6, and B12 through the expression of *SLC19A3* [46] and *TCN2* [47]. For metal ions (Fig. 2J), the Na and K channels of *ATP1A1*, *ATP1B1*, *SCNN1A*, and *SCNN1B* were mainly expressed in the jejunum and colon [48], while the transporters for bivalent metal ions like Fe, Zn, and Mn (*SLC30A10*, *SLC39A5*, *SLC39A8*) were highly expressed in the jejunum and *SLC39A7* in the jejunum and colon segments [49, 50]. The expression of *AQP3* and *AQP11* was mainly found in the jejunum and *AQP8* in the colon (Fig. 2K), supporting the results that most water was absorbed in the small intestine and further dehydration occurred in the large intestine [51].

**Fig. 3:**
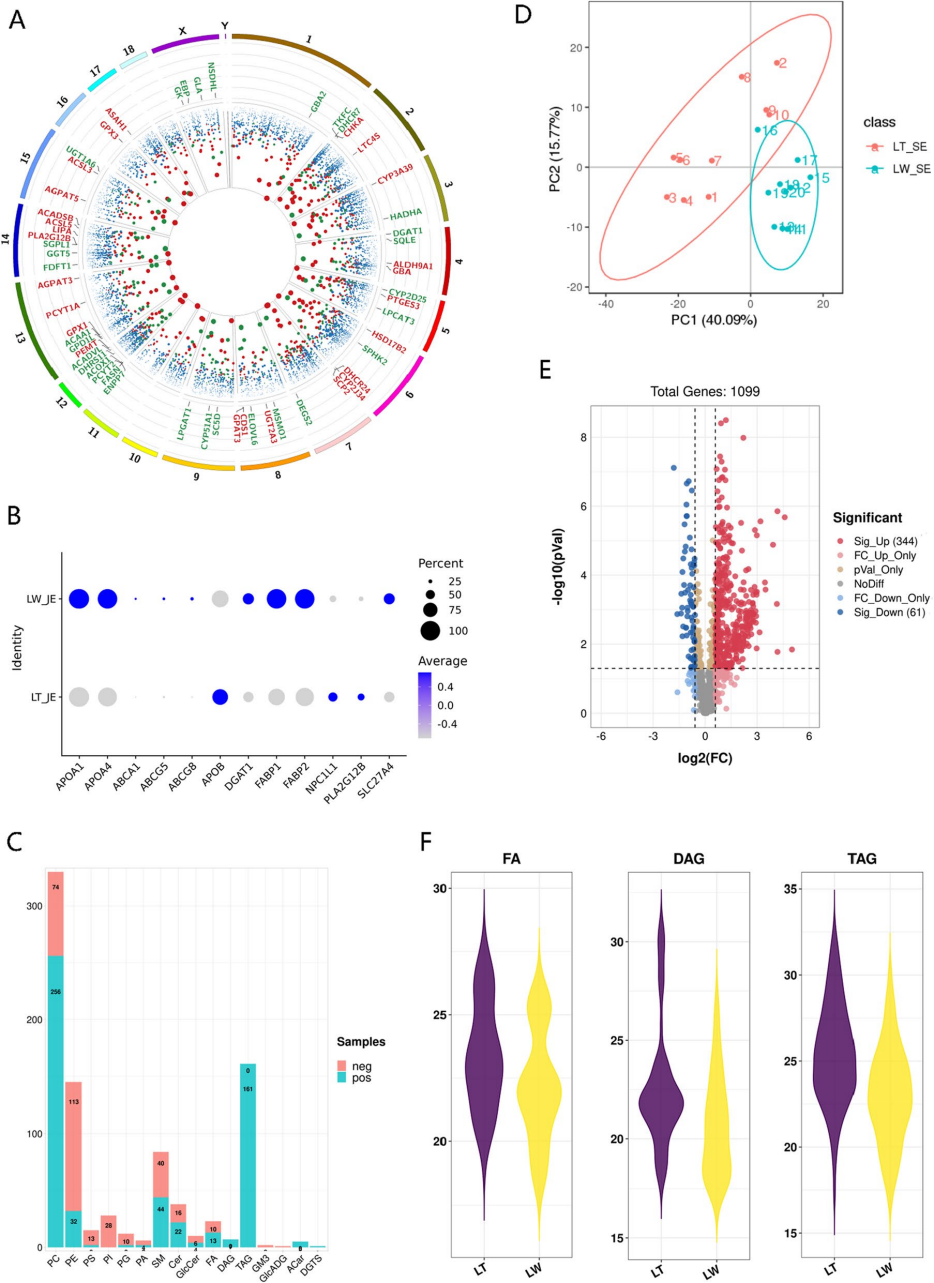
Breed-specific differences involved in lipid digestion and absorption in porcine small intestinal enterocytes. **A** Differentially expressed genes in small intestinal epithelial cells between LW and LT piglets. The outer ring represented 1 to 18 porcine autosomal and X and Y chromosomes. The middle ring indicated the differentially expressed genes involved in lipid metabolism; red symbols represented upregulated genes in LT pigs, and green represented downregulated genes. The scatter plot in the inner ring represented all genes presented in porcine enterocytes; the size of the solid circles represented for log (*Q* value, 2). **B** Expression patterns of identified genes in fat digestion and absorption enrichment in small intestinal enterocytes between LW and LT piglets. **C** Lipid metabolites identified in porcine serum. PC, phosphatidylcholine; PE, phosphatidylethanolamine; PS, phosphatidylserine; PI, phosphatidylinositols; PG, phosphatidylglycerols; PA, phosphatidic acids; SM, sphingomyelines; Cer, ceramides; GlcCer, glucosyl ceramides; FA, free fatty acids; DAG, diacylglycerol; TAG, triacylglycerol; GM3, monosialodihexosyl ganglioside; GlcADG, glucuronosyl diacylglycerol; ACar, acylcarnitine; DGTS, diacylglycerol trimethyllhomoserine. **D** PCA analysis of the identified metabolites in porcine serum between LT and LW piglets. **E** Volcano plot showing the differential lipid metabolites in porcine serum between LT and LW piglets. The red circles represented increased metabolites in LT pigs, and green represented decreased metabolites. **F** Violin plots showing FA, DAG, and TAG concentrations in porcine serum between LT and LW piglets

As small intestines show breed-specific differences, they likely play emerging roles in lipid absorption and metabolism between the indigenous fat-type and lean-type piglets [52, 53]. We therefore compared the gene expression between LT and LW piglets in small intestinal epithelial cells (Table S3A) and captured a total of 1381 DEGs (Fig. 3A), including 761 upregulated and 620 downregulated candidates in the LT breed. Kyoto Encyclopedia of Genes and Genomes (KEGG) analysis revealed that these DEGs were significantly enriched in modulating intestinal energy and nutrient metabolism (Table S3B), such as oxidative phosphorylation, glycolysis/gluconeogenesis, and fatty acid degradation in the metabolism category, as well as fat digestion and absorption at the organismal systems level. In fat digestion and absorption enrichment, the *APOB*, *NPC1L1*, and *PLA2G12B* genes were significantly up-expressed in the intestinal enterocytes of the LT breed, while the *APOA1*, *APOA4*, *ABCA1*, *ABCG5*, *ABCG8*, *DGAT1*, *FABP1*, *FABP2*, and *SLC27A4* genes were highly expressed in the LW enterocytes (Fig. 3B). The transporters of intestinal FABPs are intracellular lipid-binding proteins and display high affinity binding for long-chain fatty acids [54], and FABP1 preferentially directs fatty acid toward oxidative pathways while FABP2 directs fatty acids to triacylglycerol (TAG) synthesis [55]. In addition, small intestine-rich SLC27A4 (fatty acid transport protein 4, FATP4) is a cell-surface fatty acid transport protein for the trafficking of long-chain fatty acids [56] and exhibits acyl-CoA synthetase activity for lipid beta-oxidation [57]. In general, high activity by intestinal *FABP1*, *FABP2*, and *SLC27A4* contributed to the transport of fatty acids across the cell membrane [58]. Once intestinal lipids are digested and absorbed by the intestinal epithelium, and they mainly undergo β-oxidation for energy supply or intracellular TAG re-synthesis and subsequent translocation around the body for later use [52]. The KEGG enrichment of fatty acid degradation was most significant at the lipid metabolism level (Table S3B), and the pathway genes of *ACAA1* [59], *ACOX1* [60], *ACADVL* [61], and *HADHA* [62] encoded rate-limiting enzymes for fatty acid beta-oxidation were significantly increased in LW breed (Table S3A), strongly implying the high-energy requirements of LW piglets. In addition, the ABCA1, ABCG5, and ABCG8 mediated the secretion of cellular cholesterol and phospholipids to extracellular acceptors, APOA protein families, to form nascent high-density lipoprotein (HDL) [63, 64]. In this study, the relatively high expression of these genes was observed in the LW piglets when compared to LT piglets, and their functions suggested a panel of breed-specific advantages of the LW breed in lipid uptake, lipid cytoplasmic oxidation, and HDL biogenesis in the intestinal epithelium. In contrast, NPC1L1 was enriched in the small intestine absorptive enterocytes and transported dietary sterols to target the encoding product of the *APOB* gene, resulting in the formation of chylomicron (CM) [65, 66]. In addition, the *PLA2G12B* expression governed intestinal and hepatic cytosolic lipoprotein production and secretion, such as CM and very low-density lipoprotein (VLDL) [67, 68], and is associated with serum triglycerides (TG) level in part [69]. We further utilized lipidomic technology and subsequently detected 398 negative and 701 positive lipid metabolites in porcine serum (Table S3C), while phosphatidylcholine (PC), phosphatidylethanolamine (PE) and TG were obviously rich in the blood (Fig. 3C). The PCA analysis demonstrated that the metabolite composition between LT and LW was clearly distinguished (Fig. 3D), and a total of 405 metabolites were significantly regulated, including 344 increased and 61 decreased candidates in the LT breed (Fig. 3E). In detail, 4 fatty acids, 7 diacylglycerols, and 129 triacylglycerols were all significantly upregulated in the LT serum (Fig. 3F; Table S3D), in agreement with the high level of *APOB*, *NPC1L1*, and *PLA2G12B* expression for triglyceride-rich lipoprotein synthesis and secretion in the LT enterocytes.

**Fig. 4:**
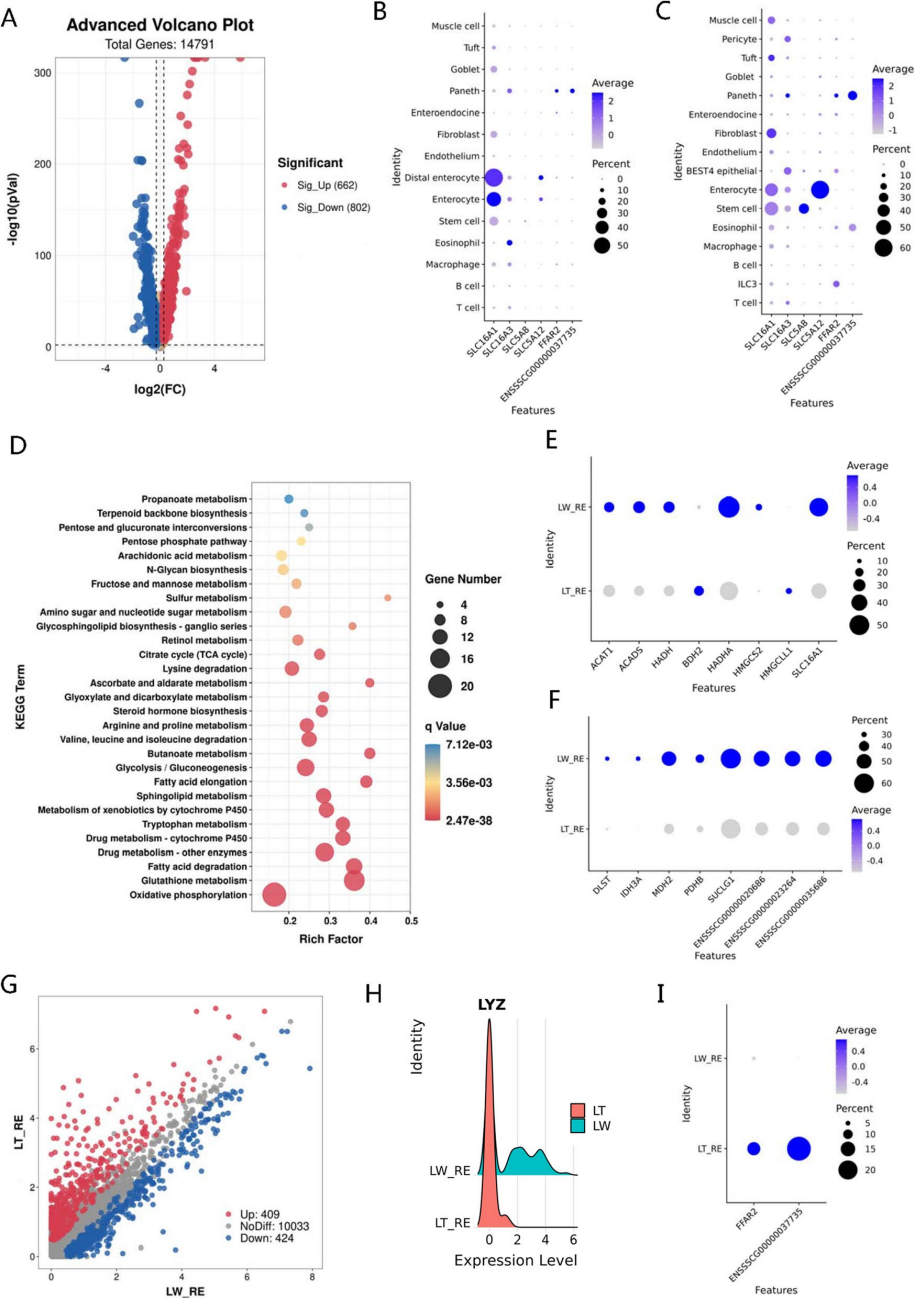
Breed-specific differences involved in SCFA functions in porcine large intestinal enterocytes and Paneth cells. **A** Volcano plot showing differentially expressed genes between LT and LW piglets in large intestinal epithelial cells. **B**, **C** Expression patterns of SCFA transport-related and receptor genes in distinct cell types from the small intestine (**B**) and large intestine (**C**), respectively. **D** KEGG functional enrichment analysis of differently expressed genes in large intestinal epithelial cells between LT and LW piglets at metabolism classification. **E**, **F** Expression patterns of β-oxidation (**E**) and TCA cycle (**F**) genes in porcine colonic enterocytes between LT and LW piglets. **G** Scatter plot showing the differentially expressed genes between LT and LW piglets in large intestinal Paneth cells. **H** Ridge plot showing the expression of LYZ gene in large intestinal Paneth cells between LT and LW piglets. **I** Dot plot of the SCFA-related receptor gene FFAR2 and HCAR2 in large intestinal Paneth cells between LT and LW piglets

The large intestine is the site of bacterial fermentation, mainly producing SCFA in monogastric animals [32], and these bacteria-derived SCFA generally contribute to maintaining intestinal homeostasis by acting not only as an energy source but also as multiple signaling modalities [70]. To reveal colonic metabolic characteristics between different breeds, we detected 1464 DEGs between LT and LW piglets in colonic enterocytes (Table S3E), and a total of 662 were upregulated and 802 were downregulated in the LT breed (Fig. 4A). The monocarboxylate transporters (MCTs) and sodium-coupled monocarboxylate transporters (SMCTs) are the dominant transmission routes for the entry of SCFA into intestinal epithelium, thereby providing energy to epithelial cells [71]. In our study, SLC16A1 (MCT1) was a major route for SCFA absorption in porcine colonic enterocytes (Fig. 4B) and was highly expressed in the LW breed (Table S3E), whereas SLC5A12 (SMCT2) showed an essential role for SCFA transfer in jejunal epithelial cells (Fig. 4C). In addition, the DEGs were significantly associated with several nutrient metabolisms (Table S3F), including butanoate metabolism and citrate cycle (Fig. 4D). In butanoate metabolism, the expressed levels of *SLC16A1*, *ACADS*, *HADH*, and *ACAT1* involved in β-oxidation were significantly increased in the LW breed (Fig. 4E), as well as the core gene of citrate synthase (CS) in the mitochondrial tricarboxylic acid (TCA) cycle (Fig. 4F). In LW piglets, an overall increase in the expression of these essential genes related to β-oxidation and TCA cycle, in particular, such as *ACADS* and *CS*, the enzymes that catalyze the rate-limiting steps of short-chain fatty acid dehydrogenation in the large intestines [72]. Our findings suggested that large-intestinal SCFA that was taken up with epithelium in the LW breed was preferentially trafficked toward oxidative pathways releasing energy, in line with high energy requirements for the rapid growth of the LW breed. In addition, over the past decade, it has been identified that a widespread receptor system exists for SCFA [73]. The SCFA-activated G-coupled protein receptors, FFAR2 and HCAR2, were significantly enriched in porcine Paneth cells in the small and large intestinal segments (Fig. 4B, C). In general, Paneth cells are specialized intestinal epithelial cells associated with autophagy activity [74] and regulate host-bacterial interactions by producing antimicrobial peptides [75]. In the Paneth cells of the LT breed, we found a total of 408 upregulated genes (Fig. 4G; Table S3G), and these candidates were significantly enriched in multiple immune-related events (Table S3H), in agreement with increased immune-related cell numbers (Table S3I) and enhanced disease resistance in LT piglets [76]. In addition, lysozyme is an innate enzyme with potent antibacterial properties mainly found in Paneth cells [77]. However, we discovered that the lysozyme gene was highly expressed in the Paneth cells of the LW breed (Fig. 4H), and by contrast, the SCFA-related G-coupled protein receptor genes were obviously downregulated in the LW breed (Fig. 4I), probably implying the alternative synthesis and secretion system of lysozyme [78].

### The catalog of metagenome-assembled genomes from porcine gut microbiota

The gut microbiota is a central regulator of host metabolism [79]. The composition and function of the gut microbiota are dynamic and affected by various genetic, nutritional, and environmental factors [80]. In this study, we examined the composition and function of the jejunal and colonic microbiota in 10 LT and 10 LW piglets. We obtained a total of 265.19 gigabases (GB) of paired-end reads and achieved an average sequencing depth of 6.63 ± 0.44 Gb (mean ± s.d.) for each sample. Approximately 11.57% ± 4.42% and 81.31% ± 15.67% of original reads from jejunal and colonic segments were considered as non-host, quality-filtered microbial sequences for further analysis (Table S4A). The reads were then assembled into scaftigs for each sample using the assembly software MEGAHIT [81], and the diversity of metagenome-assembled scaftigs varied with pig breed and intestinal location (Table S4B). In detail, a total of 68,318.90 ± 3029.54, 74,771.88 ± 13,214.48, 45,487.00 ± 10,648.78, and 21,950.60 ± 4925.64 fragments with least 500-bp length were successfully assembled in the jejunal sites of LT and LW, and the colonic sites of LT and LW, respectively. We then performed MetaGeneMark to construct a gene catalog [82], and the program predicted 681,230 non-redundant open reading frames (ORFs) using a 100-bp cutoff, with an average length of 407-bp (Table S4C). The jejunal and colonic catalogs contained 308,508 and 478,568 unigenes, respectively, and a total of 302,864 (44.46%) were complete genes, while 378,366 (55.54%) were incomplete.

**Fig. 5:**
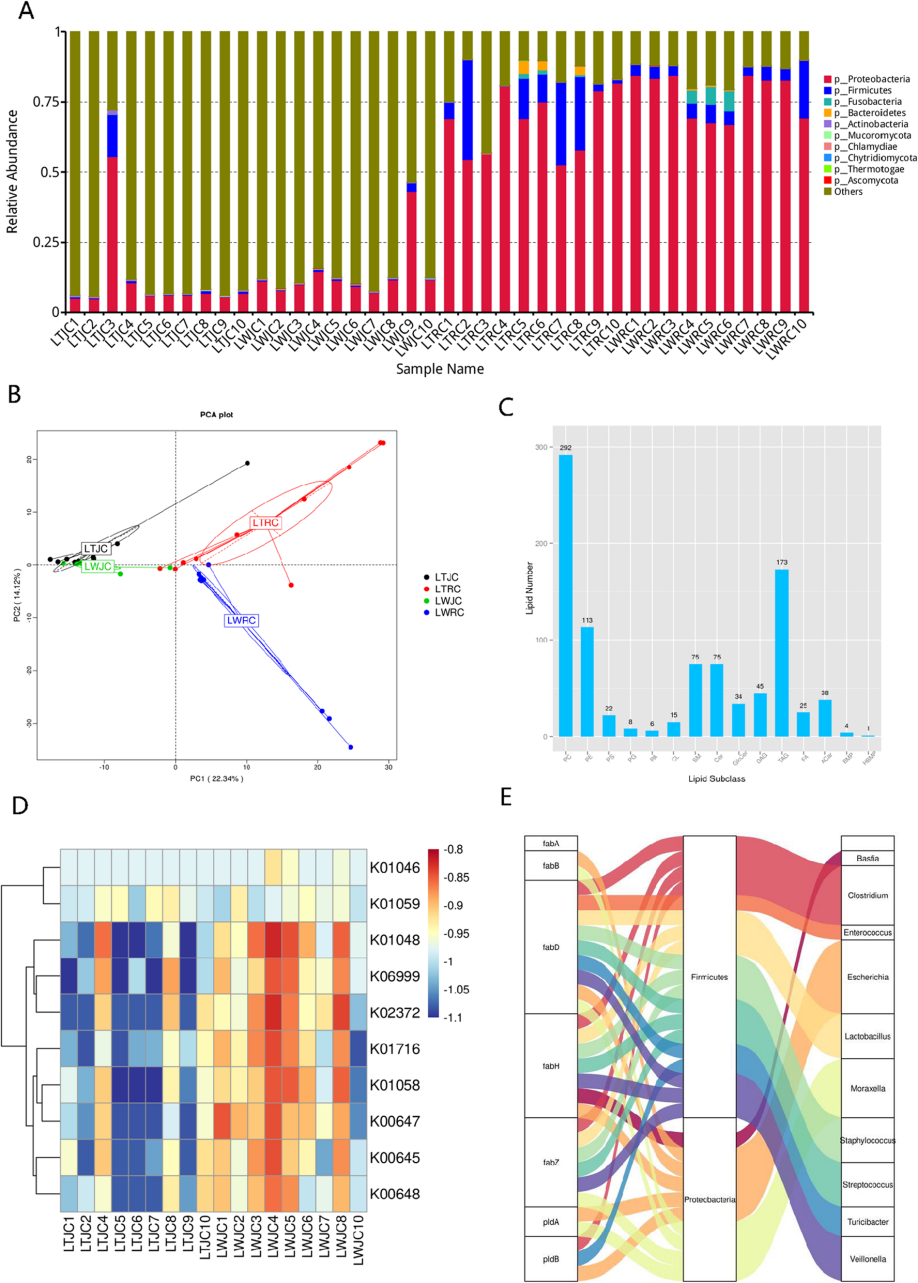
The composition, abundance, and annotation of porcine intestinal microbiota and lipidome in LT and LW piglets. **A** Bar plot showing the relative abundance of intestinal microbiota at the phylum level. **B** Principal component analysis of the tested samples at the genus level. **C** Lipid metabolites identified at positive ion mode in porcine jejunal contents. **D** Heatmap showing the gene expression levels involved in bacterial fatty acids synthesis in porcine jejunum. **E** The distribution of FabA, FabB, FabD, FabH and FabZ genes on jejunal bacteria at phylum and genus levels. The colors of the lines represented different taxonomy levels

To determine the composition of the gut microbiota, we blasted the identified genes to 3302 non-redundant references extracted from the National Center for Biological Information (NCBI), including bacteria, fungi, archaea, and viruses, and only 264,438 genes could be taxonomically classified. More than 99.86% of the classified genes were assigned to bacteria, whereas the remaining 0.13% and 0.14‰ belonged to eukaryota and archaea, respectively. These classified genes were annotated to 53 phyla, 71 classes, 149 orders, 284 families, and 861 genera. At the phylum level, Proteobacteria dominated the intestinal microbial communities of all samples (Fig. 5A), representing 8.49% ± 2.86% of the jejunum and 72.57% ± 10.88% of the colon, respectively, followed by Firmicutes, Fusobacteria, and Bacteroidetes (Table S4D). This distribution was in agreement with a previous observation [83]. At the genus level, *Escherichia*, *Neisseria*, and *Campylobacter* were the dominant phylotypes in the jejunal group, while *Escherichia*, *Clostridium*, and *Turicibacter* were the most abundant genera in the colonic group (Table S4E). The bacterial taxa detected in all tested samples were defined as the core bacteria of pig gut microbiome, and 27 (81.82% in all annotated phyla) phyla, 111 (29.68%) genera, and 143 (14.61%) species were identified as core bacteria in the jejunal samples, as well as 15 (28.30%) phyla, 60 (7.06%) genera, and 95 (2.91%) species in the colonic samples (Table S4D-F). The abundances of these 143 species in the jejunum and 95 in the colon occupied more than 97.02 ± 2.90% and 87.04% ± 12.59% of the total abundance of the annotated bacterial species, implying their high prevalence and important roles in the gut microbiome of pigs. We readily separated the tested samples with pig breed and intestinal location based on genera profiles by principal component analysis (Fig. 5B), non-metric multi-dimensional scaling analysis (Fig. S2A), or hierarchical clustering analysis (Fig. S2B). We then removed two outliers of LTJC3 and LWJC9 and explored whether some microbial candidates were significantly different in the gut microbiota between LT and LW piglets. Based on LefSe analysis [84], a total of 60 microbial candidates were estimated to be discriminative features in the jejunum (Table S4G), and 41 were inferred to be putative genomic biomarkers in the colon (Table S4H). In detail, the genera *Bacteroides*, *Deinococcus*, *Lactobacillus*, *Lactococcus*, *Neisseria*, *Campylobacter*, and *Francisella* were significantly enriched in the small intestine segment of the LT breed, whereas *Enterobacter*, *Photorhabdus*, and *Serratia* were enriched in the LW breed. In the large intestine segment, genera of *Lactobacillus*, *Niameybacter*, *Neisseria*, *Campylobacter*, and *Francisella* showed higher levels in the LT breed, as well as *Veillonella*, the species of *Enterococcus hirae* from *Enterococcus* genus, the species of *Fusobacterium russii* from *Fusobacterium* genus, and the species of *Actinobacillus succinogenes* from *Actinobacillus* genus in the LW breed.

To investigate microbial functions of porcine intestines, we annotated the metagenome-assembled genes in our catalog to the KEGG and eggNOG datasets. A total of 243,614 (35.76%) and 245,158 (35.99%) non-redundant genes were successfully annotated to 6645 KEGG orthologues (Table S5A) and 7014 eggNOG orthologues (Table S5B), respectively. A large number of KEGG-annotated genes were enriched in various metabolic processes, including carbohydrate metabolism, amino-acid metabolism, metabolism of cofactors and vitamins, nucleotide metabolism, and energy metabolism (Fig. S2C), while the eggNOG-annotated genes were associated with carbohydrate transport and metabolism, amino-acid transport and metabolism, cell membrane biogenesis, inorganic transport and metabolism, and energy production and conversion (Fig. S2D). These results were in agreement with the physiological structure and biological function of intestinal tracts in nutrient transport and metabolism. In the jejunum segment, a total of 2494 KEGG ortholog groups (KOs) were differentially presented between LT and LW piglets, and only 70 KOs were significantly enriched in the LT breed (Table S5C). At the pathway level, we detected a total of 398 functional terms annotated with metagenome-assembled genes (Table S5D), and more than 56.76% (105) functional differences were enriched in the Metabolism level, including 11 functional categories associated with lipid metabolisms, such as alpha-linolenic acid metabolism, sphingolipid metabolism, glycerophospholipid metabolism, and arachidonic acid metabolism (Table S5E), in agreement with the high sphingolipid and glycerophospholipid contents in porcine jejunum of our study. We obtained a total of 2134 metabolites with lipidomic technology in porcine jejunum, and 1585 had identification information (Table S5F). In detail, 346 molecules in the lipidome belonged to sphingolipids, including 163 ceramide and 120 sphingomyelins (SM), while 909 metabolites were primarily from glycerophospholipids, consisting of 348 phosphatidylcholine (PC), 279 phosphatidylethanolamine (PE), and 90 phosphatidylserine (PS) molecules (Fig. 5C and Fig. S2E). In general, the gut microbiota is now considered as one of the key elements contributing to the regulation of intestinal lipid metabolism and composition through interaction with the diet [85]. In our study, a large number of phospholipid metabolites were identified in the jejunum of the tested sucking piglets, and glycerophospholipids and sphingolipids were obviously enriched in the intestinal contents, in agreement with the concentration and composition of the milk lipids [86]. Comparing the metabolomic data between LT and LW piglets, there were 731 significantly changed metabolites, and 9 of 12 free fatty acids (FFAs) were highly enriched in LW groups (Table S5G). In general, a number of long-chain free fatty acid (LCFA) releases were mainly from lipid digestion and bacterial fatty acid biosynthesis in the small intestine [87, 88]. For lipid digestion, pathway analysis of KEGG orthologs found that genes for glycerophospholipid digestion via phospholipase (*K01058*, *K01048*, *K06999*) were highly enriched in LW groups, whereas the gene expression of triacylglycerol lipase (*TGL2*, *K01046*) and lipoprotein lipase (*LPL*, *K01059*) involved in lipolysis appeared no differences in comparison to LT groups (Table S5C). The results implied more obvious advantages in lipid digestion by hydrolyzing phospholipids to FFAs in the lean-meat type piglets. In recent, several data have provided pieces of evidence in animal models that milk phospholipids affect infant gastrointestinal health and development [89], and the possible mechanism to benefit from milk phospholipid may occur via microbiota-lipid interaction [90]. These biological interactions allow gut microbiota to bind dietary lipids and express lipolytic and phospholipolytic enzymes to digest milkfat [91], and these enzymes within the gut microbiome thus act like a second liver to break down and transform lipids [92]. Previous analysis indicated that the majority of phospholipase-positive strains mainly belong to the phylum Firmicutes [91], Bacteroidetes [93], Enterobacteria and Actinobacteria [94], and Proteobacteria [95]. In line with our study, Proteobacteria and Firmicutes were the predominant intestinal microbial phyla and characterized as phospholipase producers, including the genera of *Clostridium*, *Turicibacter*, *Escherichia*, and *Moraxella* (Table S5H). We observed that only the strains from *Escherichia*, dominated the intestinal microbial communities during the first week of life [96, 97], were significantly presented in LW piglets (Table S4I), and exhibited the accumulation of membrane phospholipase [98], strongly implying their biological functions in lipid accumulation of small intestinal tract in response to pig breed. For fatty acid biosynthesis, FabA (*K01716*), FabB (*K00647*), FabD (*K00645*), FabH (*K00648*), and FabZ (*K02372*), encoding to synthetases, responsible for bacterial LCFA synthesis and elongation [99], showed elevated relative abundances in the LW group (Fig. 5D). These genes that were indispensable for LCFA synthesis were mainly expressed in the genera *Staphylococcus*, *Enterococcus*, *Lactobacillus*, *Streptococcus*, *Clostridium*, *Turicibacter*, *Veillonella*, *Escherichia*, *Basfia*, and *Moraxella* (Fig. 5E; Table S5H), suggesting that they participated LCFA accumulation. In general, the hindgut microbiota has been well recognized in maintaining intestinal homeostasis and symbiosis by fermenting indigestible dietary components and thereby producing SCFA, such as acetate, butyrate, and propionate [70, 71]. The potential for SCFA production is frequently a focus of many mammalian gut microbiome studies, as even in monogastric animals like pigs, up to 25% of daily energy requirements are met by SCFAs [100]. In the pre-weaning period of piglets, breast milk oligosaccharides are indigestible to the infant and, for this reason, reach the colon intact, thereby acting as metabolic substrate necessary for intestinal microbiota to generate SCFAs that are critical for gut health [101]. In the colon segment, we identified a total of 1455 differentially regulated KOs (Table S5I), as well as 103 KEGG pathways between LT and LW groups (Table S5J). Here, we found that the SCFA-producing enzymes, namely acetate kinase (*ackA, K00925*), phosphate acetyltransferase enzymes (*pta*, *K00625*, or *K13788*), propionate CoA-transferase (*pct*, *K01026*), butyryl-CoA to acetate CoA-transferase (*bua*, *K01034*, or *K01035*) and butyrate kinase (*buk*, *K00634*, or *K00929*) [102], were mainly encoded in the Actinobacteria, Bacteroidetes, Firmicutes, Fusobacteria, and Proteobacteria phyla and widely distributed in 23 genera (Fig. S2F), and notably, *Clostridioides* encoding butyrate kinases, were significantly enriched in LT groups (Table S4J). Butyrate production by *Clostridioides* has been demonstrated in a previous report [32] and can regulate apoptosis, enhance barrier function, and reduce inflammation in the host [103], associated with the strong disease-resistance characteristic of LT pigs. In addition, the total expression levels of the above genes were further compared between LT and LW breeds, but no significant differences were found (Table S5I), the results demonstrating that there were no differences in SCFA synthesis between pig breeds, at least in the pre-weaning period. Our findings suggested an additional mechanism involved in crosstalk between the early intestinal microbiota and lipids and expanded our understanding of lipid metabolism for animal production and human health.

### The landscape of porcine liver cell types

**Fig. 6:**
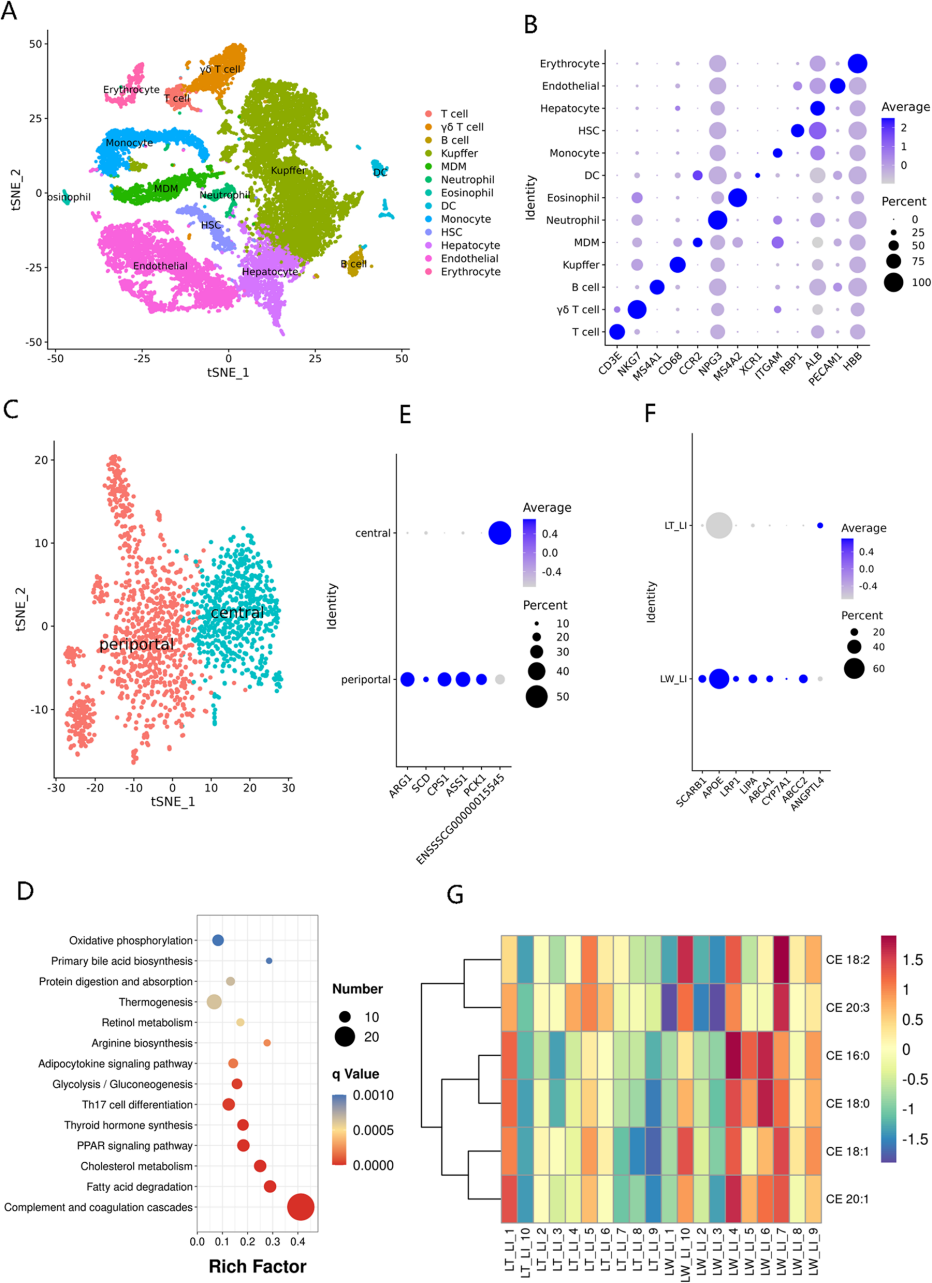
Distinct expression patterns of genes related to cholesterol metabolism and bile secretion in porcine liver tissues. **A** t-SNE analysis of 18,152 single cells from porcine liver tissues, with 13 major cell types labeled in different colors. **B** Dot plots showing the expression of representative marker genes in porcine livers. **C** Graph-based re-clustering of porcine hepatocytes revealed two subpopulations: periportal and central hepatocytes. **D** KEGG enrichment analysis of the periportal hepatocyte-specific genes. **E** Dot plots showing the expression of representative marker genes in porcine periportal and central hepatocytes. **F** Expression patterns of breed-specific genes involved in cholesterol metabolism and bile secretion at the digestive system categories in porcine periportal hepatocytes. **G** Heatmap showing the differential cholesteryl esters in porcine liver tissues between LT and LW piglets

To identify resident liver cells, we used scRNA-seq to measure the entire transcriptome of more than 18,000 dissociated liver cells. Our analysis revealed 25 distinct populations, and our atlas comprised all of the main liver cell types consisting of T cells, γδ T cells, B cells, Kupffer cells, monocyte-derived macrophages, neutrophils, eosinophils, dendritic cells, monocytes, hepatic stellate cells, hepatocytes, endothelial cells, and erythrocytes (Fig. 6A) based on a panel of landmark genes (Fig. 6B; Table S6A). In general, hepatocytes play a series of key roles in detoxification, lipolysis, and gluconeogenesis based on their zonation [104]. We therefore re-clustered 1671 hepatocytes (Table S6B) that generally showed enriched *ALB* expression (Fig. 6B) and found two diverse zonation patterns that showed dynamic differences in the gene expression profiles (Table S6C), known as periportal and central hepatocytes (Fig. 6C). KEGG enrichment analysis of the zonated genes demonstrated that periportal hepatocytes were enriched in genes responsible for biological oxidations, lipid, and cholesterol metabolism (Table S6D), including fatty acid degradation, glycolysis/gluconeogenesis, primary bile acid biosynthesis, and cholesterol metabolism (Fig. 6D), whereas central hepatocytes showed enrichment of drug metabolism genes along with numerous active immune pathways (Table S6E), based on the expression patterns of the periportal gene *ARG1*, *ASS1*, *CPS1*, *PCK1,* and *SCD* [104, 105] and the central gene *GLUL* [106] (Fig. 6E).

In general, bile acids are important physiological agents that are required for intestinal solubilization, digestion, and absorption of lipids [107], and the gut-to-liver axis plays a critical role in the regulation of bile acid synthesis, especially in the periportal hepatocytes [108]. To explore how pig breed affects hepatocytic function in lipid metabolism, we therefore compared the periportal hepatocyte transcriptomes of LT and LW piglets in our scRNA-seq datasets. We detected a total of 796 and 1143 genes that exhibited significant decreases or increases in LT groups, respectively (Table S6F). KEGG analysis on these DEGs revealed that the breed-related genes were highly enriched in the pathways responsible for energy and nutrient metabolism, and a range of significantly upregulated genes involved in cholesterol metabolism and bile secretion were observed in digestive system categories in the LW liver (Table S6G; Fig. 6F). Among them, the HDL receptor gene *SCARB1* involved in the selective uptake of HDL-associated esterified cholesterol [109], as well as the apolipoprotein E (APOE) receptor gene *LRP1* linked to hepatocytic absorption of CM remnant [110], was strongly increased in the periportal hepatocytes isolated from the LW liver, thereby improved the transfer of cholesteryl esters (CEs) from HDL or CM remnant to the hepatic cells for bile acid biosynthesis [111]. The lipid composition and metabolite profiling of porcine liver tissues were further investigated by lipidomic technology, and a total of 2202 metabolite molecules were found in our study (Table S6H), including 552 significantly regulated metabolites between LT and LW groups (Table S6I). The results showed that five CEs were all obviously upregulated in the LW liver, and CE 16:0 and CE 18:0 were significantly enriched in the LW groups (Fig. 6G), in agreement with the high level of *SCARB1* and *LRP1* expression for HDL-associated CEs and CM remnant uptake in the LW hepatocytes. Notably, blood HDL, mainly generated from the small intestine and liver tissues, is a main carrier of cholesterol in the circulation and transports excess peripheral cholesterol to the liver for bile acid synthesis [112]. The biogenesis of HDL requires APOA1 and the cholesterol transporter ABCA1 [113]. In mammals, ABCA1, a transmembrane protein that mediates the rate-controlling step in HDL particle formation, is highly expressed in the liver and intestine, and its major function is to mediate the transport of free cholesterol and phospholipids from peripheral cells to APOA1 protein for generating HDL particles [114]; deletion of intestinal ABCA1 gene in mice considerably reduces approximately 30% of the steady-state plasma HDL pool [115]. In LW hepatocytes, our survey showed that the expression levels of *ABCA1* and *APOA1* were significantly upregulated, as well as in the jejunal enterocytes of the LW breed, strongly implying the high efficiency of nascent HDL synthesis in the LW breed. For bile acid biosynthesis, lysosomal acid lipase (LIPA) has major roles in hydrolyzing CEs to cholesterol in the endocytic compartment [116], while cholesterol 7α-hydroxylase (CYP7A1), a rate-limiting enzyme of bile acid biosynthesis, contributes to catalyze the hydroxylation of cholesterol [117]. The ABCC2 generally serves as a transporter for exporting bile acids from hepatocytes to bile [118]. The expression patterns of these LW-enriched genes illustrated high efficiency in bile acid biosynthesis and secretion in the LW breed. In contrast, angiopoietin-like 4 (ANGPTL4), a secretory protein that inhibits lipoprotein lipase and modulates extracellular triacylglycerol homeostasis [119], was upregulated in the liver of LT piglets in keeping with high plasma triglyceride levels in LT piglets. A previous study has showed that bile acids influence ANGPTL4 secretion [120], in accordance with a decreased expression level of *ANGPTL4* in LW piglets and an increased level of plasmatic lipid in LT piglets. Our studies have been instrumental in elucidating the major advantages of HDL enterohepatic cycling and bile acid biosynthesis in LW piglets, and the molecular mechanisms regulating those advantages. In recent, the cholesterol contained within HDL has been extensively proven to be associated with cholesterol homeostasis and the risk of cardiovascular diseases, such as atherosclerosis in humans [121], and these key regulators and additional mechanisms delineated in our study also opened new possibilities for therapeutic interventions for the treatment of cholesterol-related diseases.

### Single-cell RNA sequencing analysis of porcine longissimus dorsi tissues

**Fig. 7:**
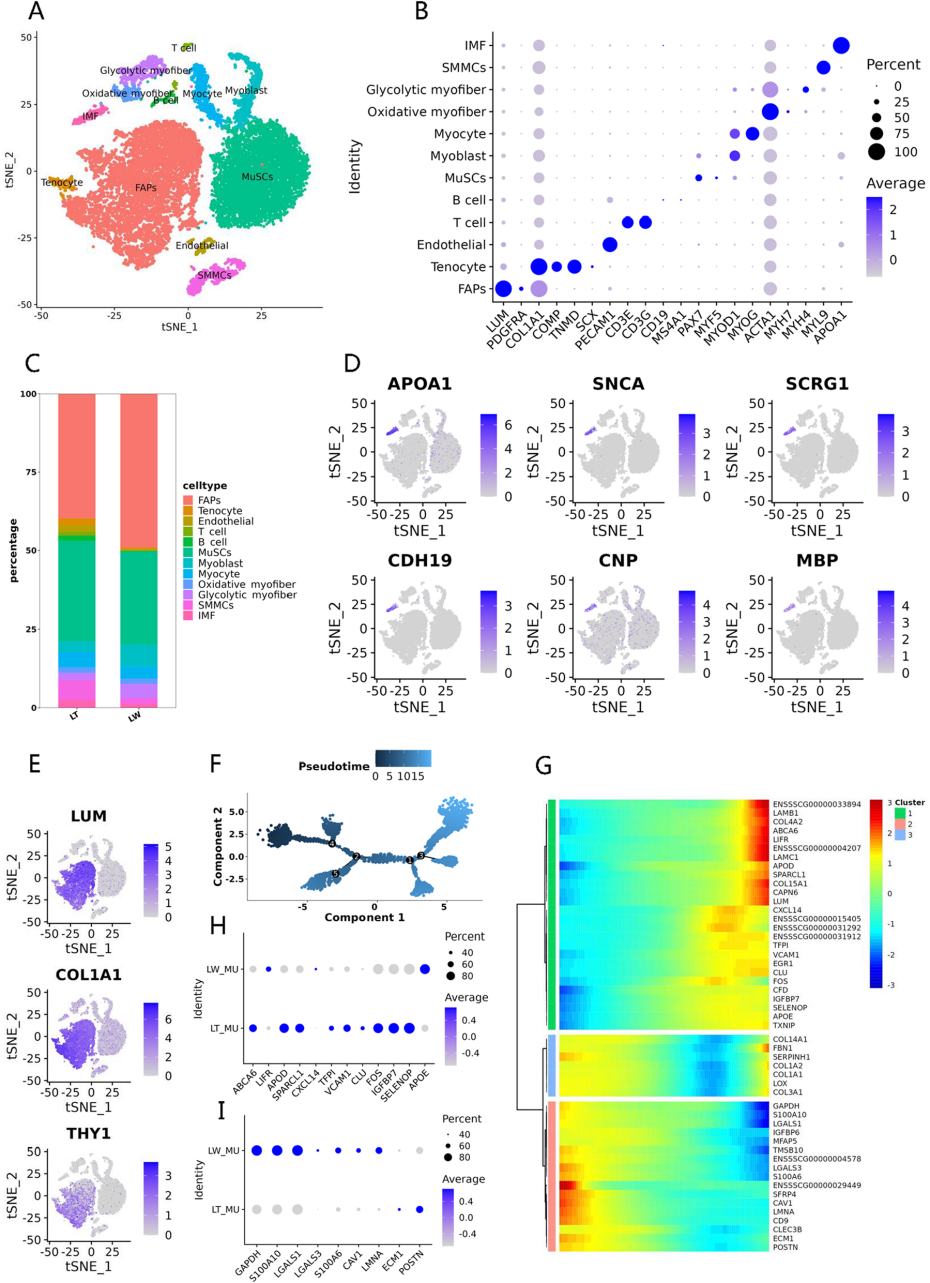
Clustering and pseudotemporal trajectories identified transcriptional dynamics of porcine muscle tissues. **A** t-SNE analysis of 19,229 single cells from porcine muscle tissues, with 12 major cell types labeled in different colors. **B** Expression of representative genes in distinct cell clusters. **C** The proportions of the 12 major cell types in porcine muscle tissues. **D** Expression of IMF-enriched genes, including APOA1, SNCA, SCRG1, CDH19, CNP, and MBP genes. **E** t-SNE plot showing the expression of LUM, COL1A1 and THY1 in porcine FAPs. **F** Pseudotime single-cell trajectory reconstructed by Monocle2 for FAP subpopulation. Pseudotime was shown in a gradient from dark to light blue. **G** Pseudotemporal heat map showing gene expression dynamics for significant marker genes. The genes (rows) were clustered into three modules, and the cells (columns) were ordered according to pseudotime. **H** The differentially expressed genes between LT and LW piglets increased along with pseudotime trajectory in porcine FAP subtypes. **I** The differentially expressed genes between LT and LW piglets decreased along with pseudotime trajectory in porcine FAP subtypes

To elucidate cell heterogeneity and dynamic breed-induced changes in porcine muscle tissues, we performed single-cell RNA sequencing on porcine longissimus dorsi tissues obtained from LT and LW piglets. We obtained a total of 19,229 single-cell transcriptomes, and further performed unsupervised shared nearest neighbor clustering, which partitioned cells into 23 groups based on their transcriptomic programs (Fig. S3A). To identify these populations, we examined the normalized expression level of canonical cell type genes (Table S7A), and revealed 12 known muscle-resident cell types (Fig. 7A), which correspond to FAPs, tenocyte-like cells, endothelial cells, T and B cells, muscle stem cells (MuSCs), myoblasts, myocytes, oxidative and glycolytic myofibers, smooth muscle cells (SMMCs), and a cluster representing IMF, based on marker gene expression (Fig. 7B). These findings are similar to those reported in other studies [122, 123].

We observed a population of myogenic progenitors, containing MuSCs and myoblasts, which expressed the myogenic transcription factors *PAX7*, *MYF5*, and *MYOD1* [124], and a population of mature myocytes, which expressed *MYOG*, involved in the regulation of myocyte fusion during myogenesis [125]. In general, the terminally differentiated muscle cells displayed distinct fiber type composition and exhibited oxidative and glycolytic metabolic phenotypes, revealing a high degree of heterogeneity within the myofiber compartment [126]. In our study, the identities of cell candidates supported by high expression of *MYH7*, encoding MyHC I, were related to oxidative fibers, while subtype with unique expression of *MYH4*, encoding MyHC Iib, contributed to glycolytic fibers (Table S7B). The glycolytic fibers within our atlas exhibited notable dynamics, and more than 4.53% (426) identified cells belonged to LW piglets, as well as 2.26% (222) in LT piglets, with the number of oxidative fibers remaining constant between two breeds (Fig. 7C; Table S7C). In muscle tissues, IMF accumulates both in (intramyocellular) and out (extramyocellular) of the muscle fibres [127]. Between cell clusters, we found a total of 658 and 1010 significantly up-expressed genes in oxidative and glycolytic myofibers, respectively (Table S7B), and KEGG annotation confirmed that the two mature myofibers were transcriptionally similar (Table S7D and E). These KEGG pathways are mainly related to energy and nutrient metabolism, such as oxidative phosphorylation, citrate cycle, pyruvate metabolism, and fatty acid degradation, consistent with biological functions of intramyocellular lipids. The intramyocellular lipids, composed mainly of triglycerides, were known to be metabolically active, providing an important energy source for muscle cells [128]. In addition, 242 (2.47%) cells revealed gene expression patterns which could be assigned to intramuscular adipocytes in LT groups, as well as 115 (1.22%) in LW groups (Fig. 7C; Table S7C). Within this cluster, cells were observed to specially express variable levels of IMF marker *APOA1* [23], while a large number of genes also preferentially detected in the clusters corresponding to IMF cells, such as *SNCA*, *SCRG1*, *CDH19*, *CNP*, and *MBP* genes (Fig. 7D), strongly suggesting them as marker candidates for intramuscular adipocytes. In addition, we observed that the 1138 IMF-specific genes had strong associations with the biosynthesis of unsaturated fatty acids and fatty acid elongation (Table S7F; Fig. S3B), including *ELOVL2*, *ELOVL5*, *ELOVL6*, *FADS1*, *FADS2*, *HSD17B1*, *HSD17B12*, *PPT2*, and *SCD*, and the co-expression modular scores of above genes were also higher in IMF (Fig. S3C). Our findings suggested that the intramuscular adipocytes were mostly associated with adipogenesis and lipid accumulation. To explore the process of lipogenesis in intramuscular, we further analyzed the transcriptomic atlas to assess how the IMF subtypes were dynamically altered in incidence and gene expression across the porcine breeds. In total, we only discerned 244 and 233 genes that exhibited significant increases or decreases in LT piglets (Table S7G), but KEGG annotation revealed no significant differences in lipid metabolism between breeds (Table S7H), including the genes of *DGAT1* and *DGAT2* (the final step in triglyceride synthesis) [129] and *acyl-CoA synthetase* family (the activation of intracellular FFA) [130]. These observations strongly supported that the IMF cells of the LT breed in muscle tissues were obviously linked to adipocytogenesis than lipogenesis processes in comparison with the LW breed.

The FAPs are the lineage precursors of specialized cells, including activated fibroblasts, adipocytes, and osteogenic cells [131]. We detected that FAP subpopulations comprised 39.76% and 49.09% of the LT and LW muscle tissues (Table S7C), consistent with human skeletal muscle, illustrating that FAPs were the most represented cell population in human skeletal muscle [132]. As expected, we found the FAP population highly expressed for *LUM* and *COL1A1*, as well as partly expressing *THY1* (*CD90*) (Fig. 7E). A previous report showed that the *THY1*− subset of FAPs was associated with adipogenesis, while *THY1*+ FAPs were involved in fibrogenesis [133]. We therefore took advantage of our scRNA-seq data and re-clustered the FAP population with high resolution, which resulted in 10 subpopulations (Fig. S3D; Table S7I). To further understand the differentiation process of FAP populations, we performed the slingshot pseudotime inference analysis to predict lineage patterns within the FAP cells (Fig. 7F), and the result indicated an origin in clusters 2, 3, and 4 (Fig. S3E), where a high proportion of *THY1*+ cells were observed (Fig. S3F). The lineage then progressed through branch point 2 to clusters 0 and 1 (Fig. S3E), consisting of a large number of *THY1*− cells and accounting for 50.65% of total identified FAPs in the LT breed (1977/3903) and 50.40% in the LW breed (2329/4621), respectively (Fig. S3F). These lines of evidences suggested that *THY1*− cells arose from *THY1*+ FAPs, and *THY1*+ cells could represent a progenitor subpopulation, in accordance with the previous study [133]. We then examined the pseudotime dynamics, which identified pseudotime-dependent genes and arranged them into clusters. This analysis revealed three gene modules, and top 50 DEGs were displayed in accordance with distinct pseudotime tracks (Fig. 7G). In detail, a series of genes, evidently increased along with the pseudotime trajectory, were significantly differential expression between LT and LW groups (Fig. 7H; Table S7J). In particular, some LT-enriched genes were predominantly involved in lipid trafficking (*ABCA6*, *APOD)* [134, 135], lipid synthesis (*FOS*) [136], lipid accumulation (*CLU*) [137], and preadipocyte differentiation (*SPARCL1*, *IGFBP7*) [138, 139], while several LW-enriched genes contributed to a significant decrease in the accumulation of triglycerides during adipogenesis (*LIFR*) [140] and lipid phagocytosis (*CXCL14*) [141]. In contrast, a number of genes seemed to lose expression along from *THY1*+ cells to *THY1*− cells, including the significantly upregulated genes in the LW group such as *GAPDH*, *S100A10*, *LGALS1*, *LGALS3*, *S100A6*, *CAV1*, and *LMNA*, as well as the *ECM1* and *POSTN* genes enriched in the LT group (Fig. 7I; Table S7J). Among them, some genes highly expressed at the beginning points of pseudotime trajectory were associated with adipocyte differentiation and adipogenesis (*LGALS1*, *POSTN*) [142, 143], lipoprotein transcytosis and lipid droplet formation (*CAV1*) [144, 145], and fat loss (*LMNA)* [144]. The results illustrated that pseudotime analysis established a basis for exploring gene candidates of FAP differentiation programs, and these breed-responsive genes that related to lipid metabolism maybe resulted in the functional differences in adipocytogenesis between LT and LW piglets.

The muscle tissues mainly rely on plasma TGs as an important source of fatty acids for subsequent oxidation or storage, and plasma TGs are generally packaged into the TG-rich CM and VLDL synthesized by the intestine and liver, respectively [146]. In our study, a significantly higher level of serum TGs was found accordingly in LT piglets, and in agreement with previous observations, similar results were identified in other Chinese native fatty breeds, such as Jinhua pigs [147], Erhualian pigs [148], and Meishan pigs [149]. The high TG concentration in local Chinese pigs illustrated that these breeds might have a lower ability to clear circulating chylomicron-TG. In the postprandial state, the clearance of circulating chylomicrons is largely dependent on hepatocyte, myocyte, and adipocyte LPL activities [150]. In general, LPL secreted by different cells is trafficked across capillary endothelial cells and anchored to the capillary wall by GPIHBP1, where it then hydrolyzes TG to release FFA [151]. The GPIHBP1 protein, an endothelial cell transporter for TG-rich lipoprotein lipase, is mainly located in capillary endothelial cells [152]. In our study, a total of 223 (2.27%) cells exhibited gene expression pattern of endothelial cells in LT groups, as well as only 29 (0.31%) in LW groups (Table S7C), and we found that the *GPIHBP1* gene was enriched in the endothelial cells (Table S7B) and significantly upregulated in the LT piglets compared to LW piglets (Table S7K), as well as the expression levels of *CD36* and *FABP5* genes in endothelial cells (Fig. S3G). The CD36 protein, located on various cell membranes, transports fatty acids in response to dietary fat [153], while *FABP5* expression is most prominent in the epidermis and considered a transporting and binding agent for lipid homeostasis [154]. These results demonstrated that the muscular differences in lipid metabolism, probably linked to pig breed, were associated primarily with the physiological functions of epithelium in TG-rich lipoprotein hydrolysis and FFA absorption.

In conclusion, we provided a comprehensive resource describing the cell dynamics and transcriptomic profiles involved in lipid metabolism in the porcine jejunum, colon, liver, and longissimus dorsi muscle, as well as the breed-specific differences in gut microbiota composition responsible for dietary fat metabolism. Our findings could open a new avenue to understanding several molecular characteristics of lipid digestibility, absorption, conversion, and deposition across tissues, which may be particularly useful for improving pork quality and developing therapies for human lipid metabolic diseases.

## Material and methods

### Animals and samples

This study included 10 Chinese fat-type piglets of LT and 10 typical lean-type piglets of LW. All piglets were collected from a large-scale pig breeding farm (Xinfeng County, Shaoguan City, Guangdong Province, China). One healthy and purebred LT or LW male piglet per litter was slaughtered on day 3 postnatal, and samples of the jejunum, colon, liver, and longissimus dorsi muscle tissues were freshly collected and immediately subjected to single-cell isolation. The jejunum digesta, colon content, serum, liver, and longissimus dorsi muscle in each piglet were harvested separately and snap-frozen in liquid nitrogen for further analysis.

### Preparation for scRNA-seq

The jejunum, colon, liver, and longissimus dorsi isolated from 10 experimental individuals in each breed were pooled and stripped of non-purpose tissues, such as blood stains, muscle, and fatty layers. Next, the harvested tissues were cut into 1 mm^3^ pieces on ice and dissociated into single cells in a dissociation solution (0.35% collagenase I, 2 mg/mL papain, 120 units/mL Dnase I) in a 37 °C water bath with shaking for 20 min at 100 rpm. Digestion was terminated with 1 × PBS (Hyclone, Logan, UT) containing 10% fetal bovine serum (Life Technologies, Foster City, CA), followed by a filtration step through 70 µm and 30 µm strainer (Miltenyi Biotec, Bergisch Gladbach, Germany). Digested cells were centrifuged at 300 × g for 5 min at 4 °C and then resuspended in 100µL 1 × PBS (0.04% BSA) and added with 1 mL 1 × red blood cell lysis buffer (MACS 130-094-183) and incubated on ice for 10 min. After incubation, the suspension was centrifuged at 300 × g for 5 min at 4 °C and resuspended in 100 μL Dead Cell Removal MicroBeads (MACS 130-090-101) and removed dead cells using Miltenyi Dead Cell Removal Kit (MACS 130-090-101). The overall cell viability was confirmed by trypan blue exclusion (Thermo Fisher Scientific, Waltham, MA), which needed to be above 85%, while single-cell suspensions were counted using a Countess II Automated Cell Counter (Thermo Fisher ScientifiC) and further diluted to a concentration of 700–1200 cells/μL for 10 × Genomics sequencing. Live cells were loaded to 10 × chromium to capture approximately 5000 single cells by 10 × Genomics Chromium Single-Cell v3 kit (10 × Genomics, San Francisco, CA) following the manufacturer’s protocol. The libraries were multiplexed and sequenced on NovaSeq 6000 sequencing system (paired-end multiplexing run, 150 bp) (Illumina, San Francisco, CA) at a minimum depth of 20,000 reads per cell.

### scRNA-seq computational analysis

Sequencing results were demultiplexed and converted to FASTQ format using Illumina bcl2fastq software v2.20 (https://support.illumina.com). Sample demultiplexing, barcode processing, and single-cell 3′ gene counting were carried out using the cell ranger pipeline (https://www.10xgenomics.com/support), and sequencing reads were aligned to *Sscrofa11.1* reference genome to obtain the raw digital gene expression matrix with the unique molecular identifier (UMI) counts per gene per cell. The cell ranger outputs were then loaded into Seurat v4.0.3 [155], and the cell candidates were removed if they expressed fewer than 500 unique genes, more than 50,000 UMI counts, or greater than 25% mitochondrial reads. To visualize the data, we further reduced the dimensionality of all high-quality cells using Seurat, and applied the t-SNE algorithm to project the cells into 2D space. In detail, the “normalization” function in the Seurat package was used to calculate the expression levels of genes; the “FindClusters” function with default parameters was performed to perform cell clustering; the “FindAllMarkers” function was used to determine the DEGs or marker genes with |Log(Fold ChangE)|> 0.25 and *P* value < 0.05. GO and KEGG annotation of DEGs were performed by the “enrichGO” and “enrichKEGG” functions in the clusterProfiler package v4.0.5 [156].

### Metagenomic library construction and quality control

The frozen contents of the jejunum and colon were thawed on ice, and total genomic DNAs were extracted by a Quick Gel Extraction Kit (Qiagen, Hilden, Germany) as described in the manufacturer’s protocol. We then measured the DNA concentration and purity with a NanoDrop ND-1000 spectrophotometer (NanoDrop Technologies, Wilmington, DE). The total DNAs were subjected to library construction through DNA-fragmentation, end-repair, adapter-ligation, and unbiased PCR amplification using NEBNext Ultra II DNA Library Prep Kit (New England Biolabs, Ipswich, MA). Agilent 2100 Bioanalyzer (Agilent Technologies, Palo Alto, CA) was used for quality control of the DNA libraries, and the qualified libraries were sequenced with an Illumina platform.

### Bioinformatics analysis of metagenome

Readfq v8 (https://github.com/cjfields/readfq) was used for preprocessing raw data, and then, clean reads were blasted with the *Sscrofa11.1* reference to filter out fragments that came from the host genome by Bowtie2 software v2.2.4 (http://bowtie-bio.sourceforge.net/bowtie2/index.shtml). MEGAHIT software v1.0.4 was further used for assembly analysis of the filtered clean data, and Scaftigs without *N* were obtained by breaking the resulting Scaffolds from the *N* junction [157]. With the default parameters, MetaGeneMark v3.05 (http://topaz.gatech.edu/GeneMark/) was applied to perform ORF prediction for Scaftigs (≥ 500 bp) in each library, and the candidates with a length less than 100nt in the prediction results were filtered out [158]. For the ORF prediction results, CD-HIT software v4.5.8 (http://www.bioinformatics.org/cd-hit/) was used to eliminate redundancy and obtain the non-redundant initial gene catalog. The clean reads in each library were then aligned to the initial gene catalog using Bowtie2 and calculated the abundance of identified genes, while genes with reads ≤ 2 in each sample were removed to finally determine the gene catalog (Unigenes) for subsequent analysis [159]. DIAMOND software v2.9.10 (https://github.com/bbuchfink/diamond/) was used for the alignment of Unigene sequences with those of bacteria, fungi, archaea, and viruses extracted from NCBI-nr database (https://www.ncbi.nlm.nih.gov/) with *e* value < 1e − 5 [160]. Gene functional annotations were made by BLASTP search (*e* value < 1e − 5) with KEGG (http://www.kegg.jp/keeg/), eggNOG (http://eggnog5.embl.de/#/app/home), and CAZy (http://www.cazy.org/) databases.

### Lipid extraction

About 0.75 mL of methanol was added to 100 μL serum (100 mg tissue powder) in a glass tube with a Teflon-lined cap and vortexed the tubes well. Then, 2.5 mL of Methyl Tert-Butyl Ethe (MTBE) was mixed and incubated for 1 h at room temperature in a shaker. Phase separation was induced by adding 0.625 mL of MS-grade water, and the tested samples were centrifuged at 1000 × g for 10 min. The upper (organic) phase was collected, while the lower phase was re-extracted with 1 mL of MTBE/methanol/water mixture (10:3:2.5; v/v/v) and centrifuged to collect the upper phase. Combined organic phases were dried and dissolved in 100 μL of isopropanol for LC-MS/MS analysis.

### UHPLC-MS/MS analysis

All analyses were performed on a Vanquish UHPLC system (Thermo Fisher) coupled with an Orbitrap Q Exactive HF mass spectrometer (Thermo Fisher). The raw data generated by UHPLC-MS/MS were processed using the Compound Discoverer (Thermo Fisher) to perform peak alignment, peak picking, and quantitation for each metabolite. After that, peak intensities were normalized to the total spectral intensity, and the normalized data was further used to predict the molecular formula based on additive ions, molecular ion peaks, and fragment ions. Then, peaks were matched with the Lipidmaps and Lipidblast database to obtain accurate qualitative and relative quantitative results. We applied univariate analysis (*t* test) to calculate the statistical significance (*P* value). The metabolites with VIP > 1 and *P* value < 0.05 and fold change ≥ 2 or ≤ 0.5 were considered to be differential metabolites between LT and LW.

### Supplementary Information

**Additional file 1: Fig. S1.** Identification of distinct cell populations in porcine small and large intestines. A t-SNE analysis of 10,227 single-cell RNA-sequencing cells in the porcine jejunal segment, with 16 major cell types labeled in different colors. B t-SNE analysis of 12,906 single-cell RNA-sequencing cells in the porcine colonic segment, with 14 major cell types labeled in different colors. C, D Violin plots showing the representative genes of ILC3 cells (C) and pericytes (D) in porcine jejunum. E Violin plots showing the representative genes of distal mature enterocytes in the porcine colon. F dot plots showing the expression of representative markers for intestinal epithelial cells. G graph-based clustering and t-SNE plot of porcine epithelial cells revealed two subpopulations: proximal and distal enterocytes. H, I Expression of representative genes between proximal and distal enterocytes by t-SNE plot (H) and dot plot (I).

**Additional file 2: Fig. S2.** Taxonomic annotation of porcine intestinal microbiota and lipidomic analysis of porcine intestinal contents. A Non-metric multi-dimensional scaling analysis (NMDS) of the tested samples at the genus level. B Hierarchical clustering analysis of the tested samples at the genus level. On the left were the Bray-Curtis distances between different samples at the genus level, and on the right was the relative abundance distribution map of each sample at the genus level. C The KEGG ortholog annotation of the metagenome-assembled genes in porcine intestinal tracts. D The eggNOG ortholog annotation of the metagenome-assembled genes in porcine intestinal tracts. E Lipid metabolites identified at negative ion mode in porcine jejunal contents. F The distribution of the metagenome-assembled genes involved in SCFA synthesis on colonic bacteria at phylum and genus levels. The colors of the lines represented different taxonomy levels.

**Additional file 3: Fig. S3.** The single-cell landscapes and functional annotation of porcine muscle-resident cell types. A tSNE plot showing the distribution of the muscle-resident clusters in LT and LW piglets. B KEGG pathway analysis of IMF-specific genes. C The gene set scores were calculated across various muscle-resident cell types based on the AddModuleScore method. D Graph-based re-clustering of porcine FAPs revealed 10 clusters that were labeled in different colors. E Pseudotime trajectory analysis corresponds to the differentiation of the FAP subpopulation from THY1-positive cells to THY1-negative cells. The cells were colored by cluster types. F Dot plots showing the expression of LUM, COL1A1 and THY1 genes in each cluster of the FAP subpopulation. G The expression levels of CD36 and FABP5 genes in porcine endothelial cells between LT and LW piglets.

**Additional file 4: Table S1A.** The list of canonical cell type-specific markers. **Table S1B** The cluster-specific genes identified for 40 major cell clusters.

**Additional file 5:** **Table S2A.** The proportions of the 28 major clusters in porcine intestinal tracts. **Table S2B** List of canonical cell type-specific markers in porcine intestinal tracts. **Table S2C** The proportions of the 14 major cell types in porcine intestinal tracts. **Table S2D** The enterocyte-enriched genes identified in porcine intestinal tracts. **Table S2E** KEGG analysis of the enterocyte-enriched genes identified in porcine intestinal tracts.

**Additional file 6: Table S3A.** Differentially expressed genes between LT and LW piglets in small intestinal epithelial cells. **Table S3B** KEGG analysis of differently expressed genes between LT and LW piglets in small intestinal epithelial cells. **Table S3C** Lipid metabolites identified in porcine serum. **Table S3D** Differential lipid metabolites in porcine serum between LT and LW piglets. **Table S3E** Differentially expressed genes between LT and LW piglets in large intestinal epithelial cells. **Table S3F** KEGG analysis of differently expressed genes between LT and LW piglets in large intestinal epithelial cells. **Table S3G** Differentially expressed genes between LT and LW piglets in large intestinal Paneth cells. **Table S3H** KEGG analysis of differently expressed genes between LT and LW piglets in large intestinal Paneth cells. **Table S3I** The cell composition of porcine colon tissues.

**Additional file 7: Table S4A.** Quality control and preprocessing of metagenomic datasets. **Table S4B** Basic statistics of the metagenome-assembled scaftigs. **Table S4C** Basic statistics of the gene catalogues. **Table S4D** The relative abundance of intestinal microbiota at the phylum level. **Table S4E** The relative abundance of intestinal microbiota at the genus level. **Table S4F** The relative abundance of intestinal microbiota at the species level. **Table S4G** Comparison of the classification of jejunal microbiota between two breeds by linear discriminant analysis effect size (LefSe) method. **Table S4H** Comparison of the classification of colonic microbiota between two breeds by linear discriminant analysis effect size (LefSe) method. **Table S4I** List of genus differentially abundant in porcine jejunum between LT and LW samples. **Table S4J** List of genus differentially abundant in porcine colon between LT and LW samples.

**Additional file 8: Table S5A.** Functional profiles of the metagenome-assembled genes with KEGG ortholog annotation in porcine intestinal tracts. **Table S5B** Functional profiles of the metagenome-assembled genes with eggNOG ortholog annotation in porcine intestinal tracts. **Table S5C** List of KEGG orthologs differentially abundant in porcine jejunum segment between LT and LW samples. **Table S5D** Functional terms annotated with KEGG pathway in porcine jejunal segment. **Table S5E** List of KEGG pathways differentially abundant in porcine jejunal segment between LT and LW samples. **Table S5F** Lipid metabolites identified in porcine jejunal contents. **Table S5G** Differential lipid metabolites in porcine jejunal contents between LT and LW piglets. **Table S5H** The distribution of the metagenome-assembled genes on bacteria at different taxonomy levels. **Table S5I** Functional terms annotated with KEGG pathway in porcine colonic segment. **Table S5J** List of KEGG pathways differentially abundant in porcine colonic segment between LT and LW samples.

**Additional file 9: Table S6A.** List of canonical cell type-specific markers in porcine liver tissues. **Table S6B** The proportions of the 13 major cell types in porcine liver tissues. **Table S6C** The zonation-specific genes in porcine periportal and central hepatocytes. **Table S6D** KEGG analysis of the periportal hepatocyte-specific genes. **Table S6E** KEGG analysis of the central hepatocyte-specific genes. **Table S6F** Differentially expressed genes between LT and LW piglets in porcine periportal hepatocytes. **Table S6G** KEGG analysis of differentially expressed genes in porcine periportal hepatocytes between LT and LW piglets. **Table S6H** Lipid metabolites identified in porcine liver tissues. **Table S6I** Differential lipid metabolites in porcine liver tissues between LT and LW piglets.

**Additional file 10: Table S7A.** List of canonical cell type-specific markers in porcine muscle tissues. **Table S7B** The cell type-specific genes identified in porcine muscle. **Table S7C** The proportions of the 12 major cell types in porcine muscle tissues. **Table S7D** KEGG analysis of the significantly up-expressed genes in oxidative myofibers. **Table S7E** KEGG analysis of the significantly up-expressed genes in glycolytic myofibers. **Table S7F** KEGG analysis of the significantly up-expressed genes in intermuscular fat cells. **Table S7G** Differentially expressed genes between LT and LW piglets in porcine IMF subtypes. **Table S7H** KEGG analysis of differentially expressed genes in porcine IMF subtypes between LT and LW piglets. **Table S7I** The expression levels of identified genes in each cluster of FAP subpopulation. **Table S7J** Differentially expressed genes in porcine FAP subtypes between LT and LW piglets. **Table S7K** Differentially expressed genes in porcine endothelial cells between LT and LW piglets.

## Data Availability

The raw sequences were deposited into Sequence Read Archive (SRA) database with the BioProject accession number PRJNA1019009 (https://dataview.ncbi.nlm.nih.gov/object/PRJNA1019009?reviewer=vqi08r93cjqd1vmi3f994tik79).
