# Supplementary figures and images for: Integrated meta-omics reveals the regulatory landscape involved in lipid metabolism between pig breeds
Source: Microbiome. 2024 Feb 20;12:33. doi: 10.1186/s40168-023-01743-3 (PMC10877772; doi:10.1186/s40168-023-01743-3)

A

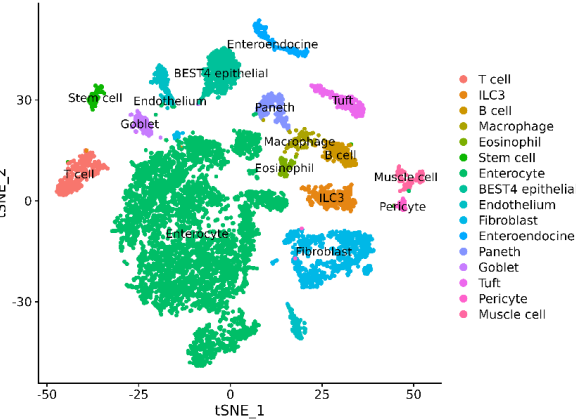

B

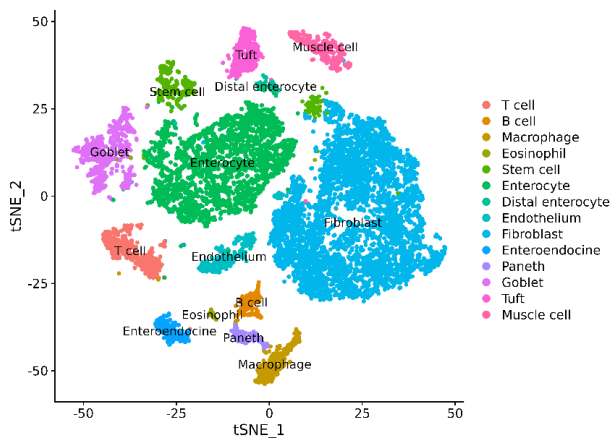

C

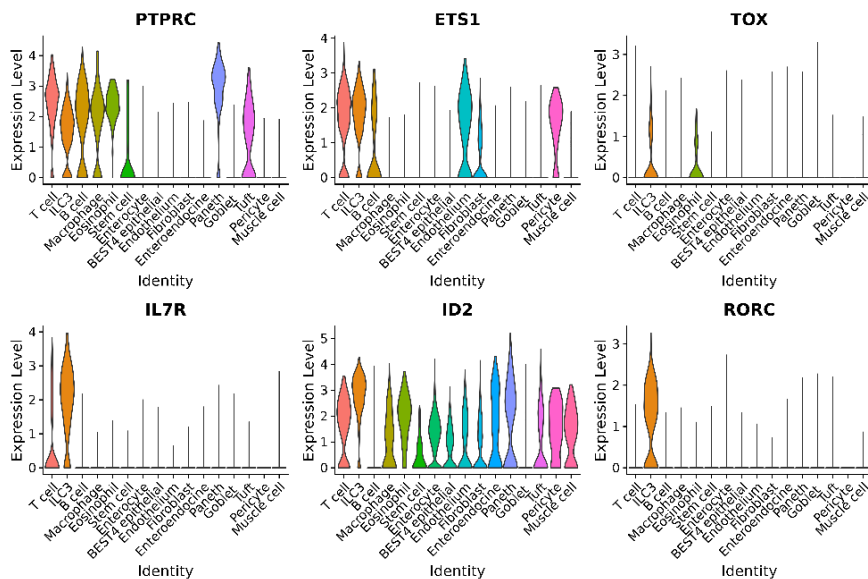

D

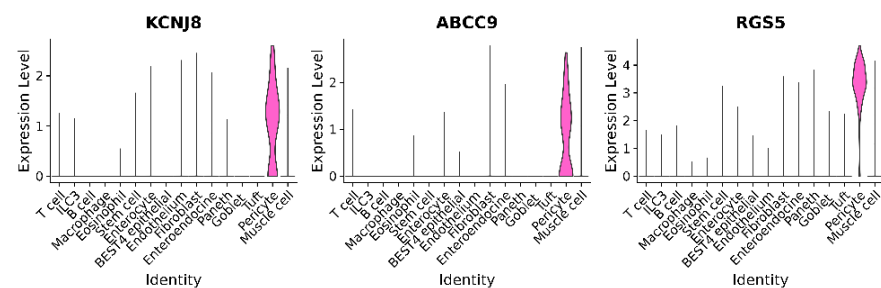

E

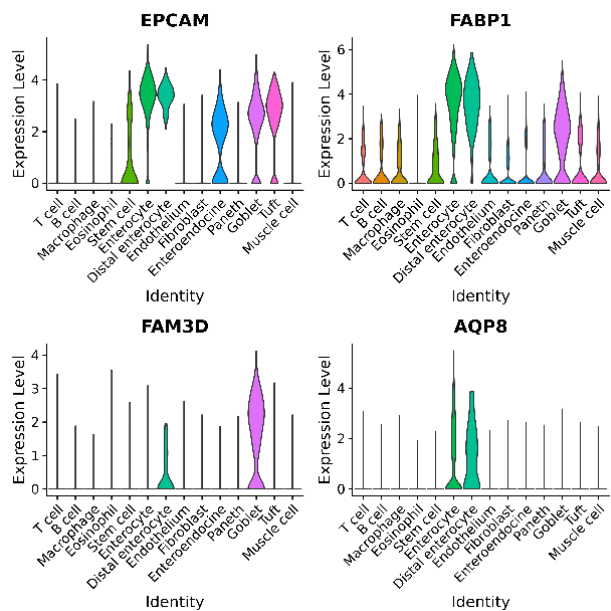

F

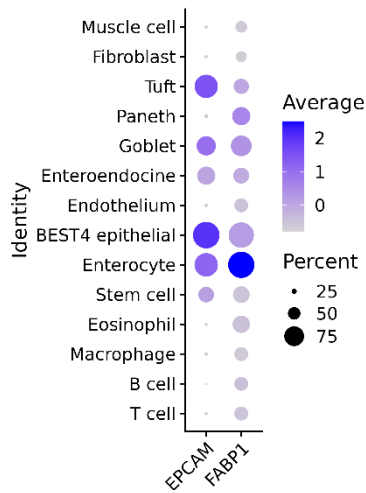

G

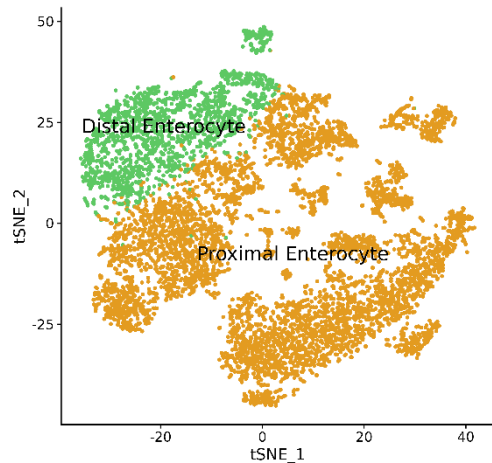

H

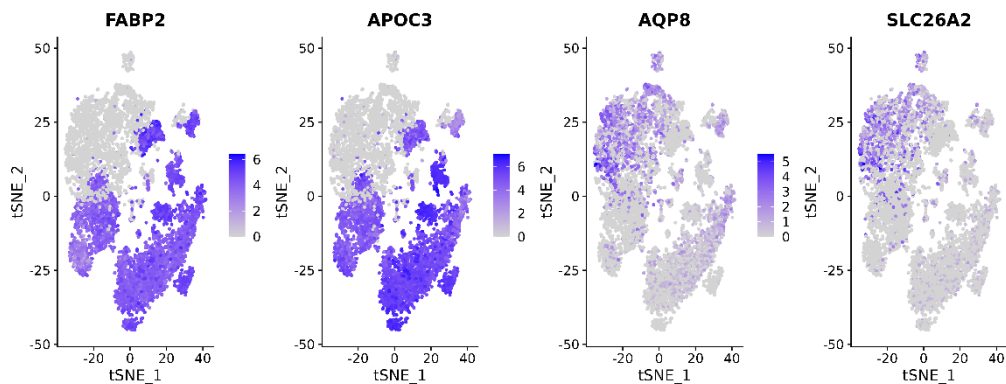

I

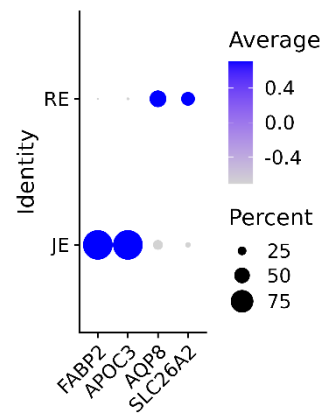

Supplement: Supplementary file 2 — Additional file 1: Fig. S1. Identification of distinct cell populations in porcine small and large intestines. A t-SNE analysis of 10,227 single-cell RNA-sequencing cells in the porcine jejunal segment, with 16 major cell types labeled in different colors. B t-SNE analysis of 12,906 single-cell RNA-sequencing cells in the porcine colonic segment, with 14 major cell types labeled in different colors. C, D Violin plots showing the representative genes of ILC3 cells (C) and pericytes (D) in porcine jejunum. E Violin plots showing the representative genes of distal mature enterocytes in the porcine colon. F dot plots showing the expression of representative markers for intestinal epithelial cells. G graph-based clustering and t-SNE plot of porcine epithelial cells revealed two subpopulations: proximal and distal enterocytes. H, I Expression of representative genes between proximal and distal enterocytes by t-SNE plot (H) and dot plot (I). [file 40168_2023_1743_MOESM1_ESM.pdf]

A

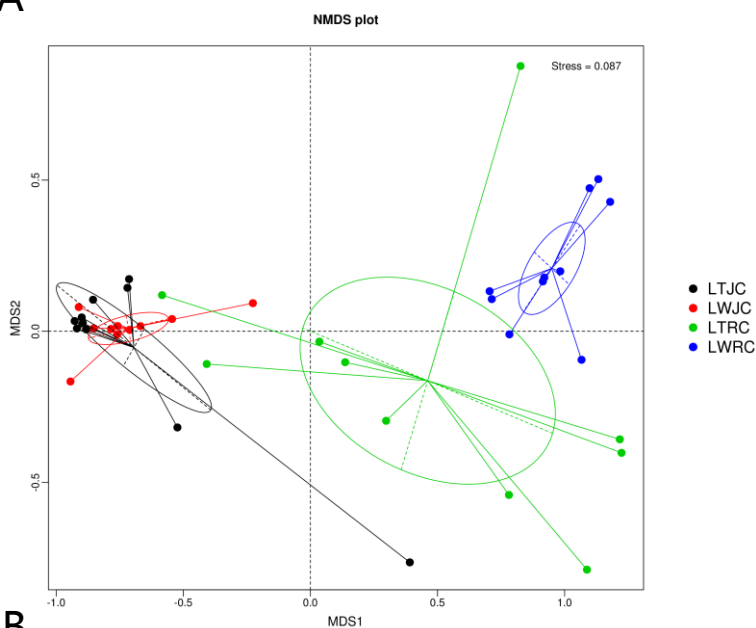

B

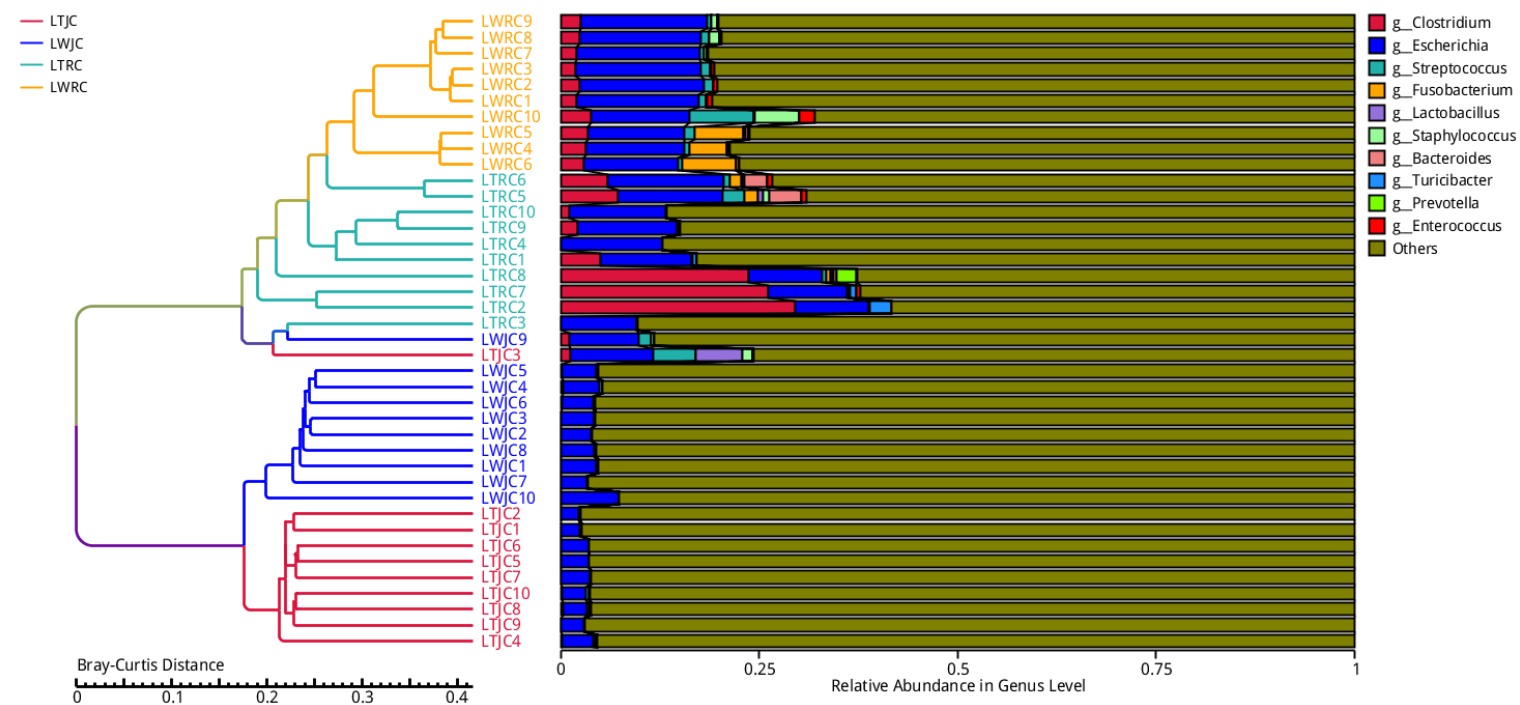

D

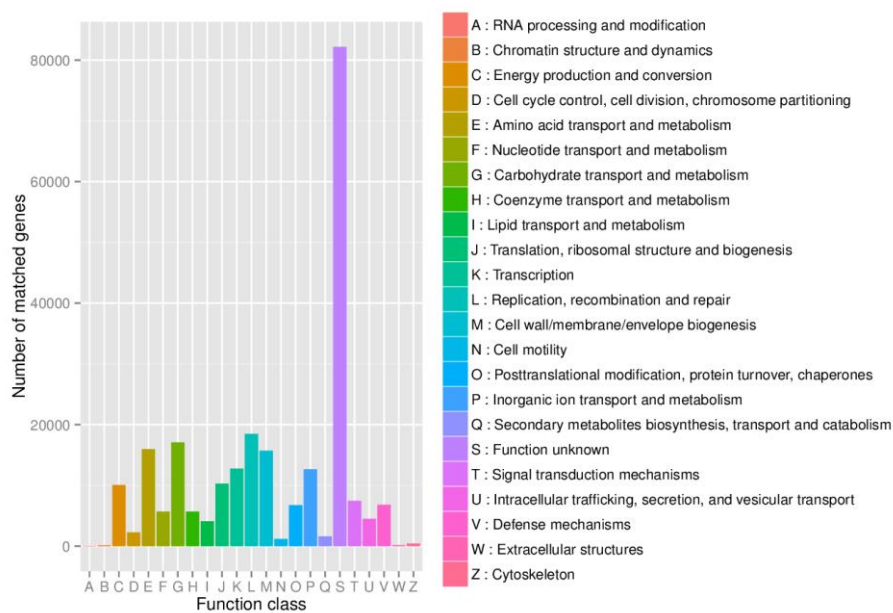

C

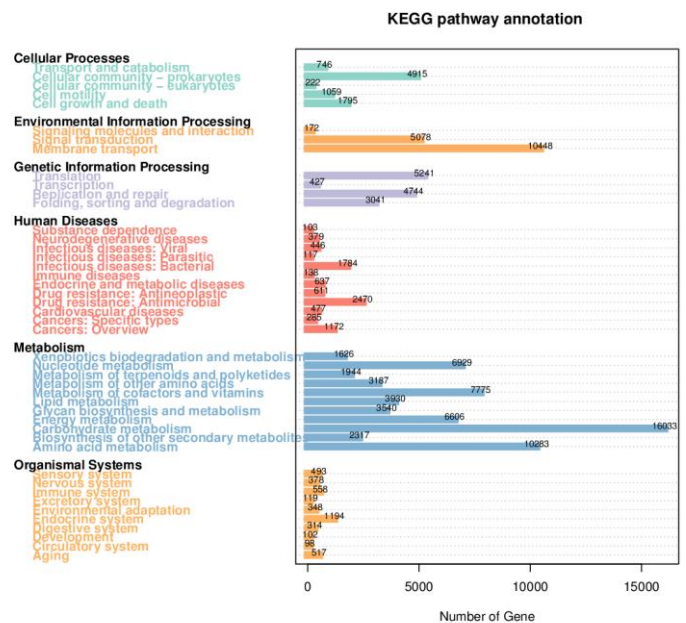

E

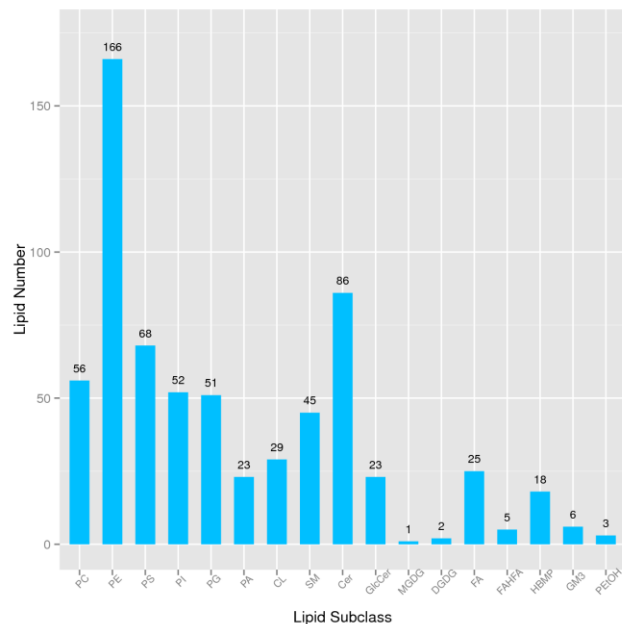

F

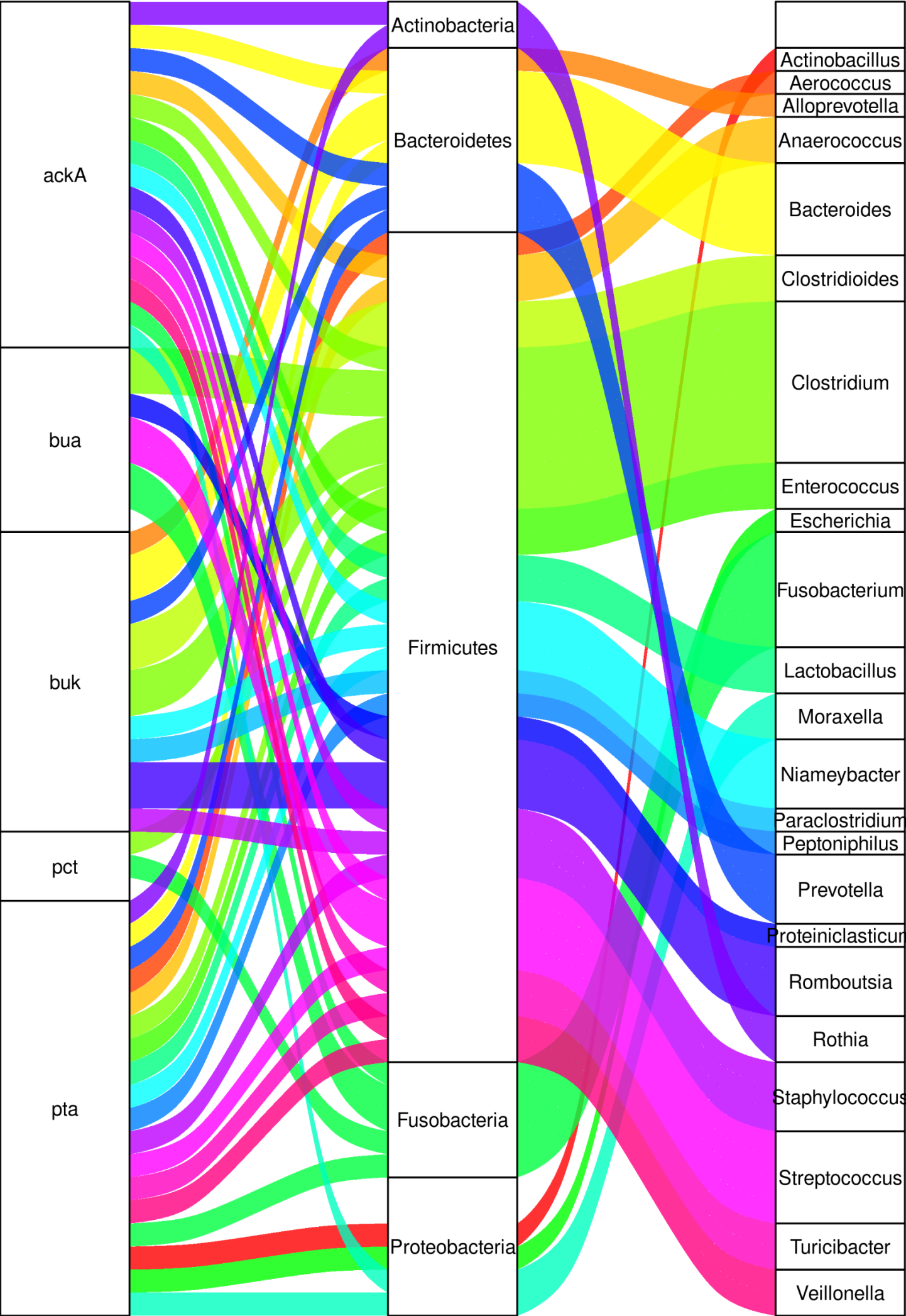

Supplement: Supplementary file 3 — Additional file 2: Fig. S2. Taxonomic annotation of porcine intestinal microbiota and lipidomic analysis of porcine intestinal contents. A Non-metric multi-dimensional scaling analysis (NMDS) of the tested samples at the genus level. B Hierarchical clustering analysis of the tested samples at the genus level. On the left were the Bray-Curtis distances between different samples at the genus level, and on the right was the relative abundance distribution map of each sample at the genus level. C The KEGG ortholog annotation of the metagenome-assembled genes in porcine intestinal tracts. D The eggNOG ortholog annotation of the metagenome-assembled genes in porcine intestinal tracts. E Lipid metabolites identified at negative ion mode in porcine jejunal contents. F The distribution of the metagenome-assembled genes involved in SCFA synthesis on colonic bacteria at phylum and genus levels. The colors of the lines represented different taxonomy levels. [file 40168_2023_1743_MOESM2_ESM.pdf]

A

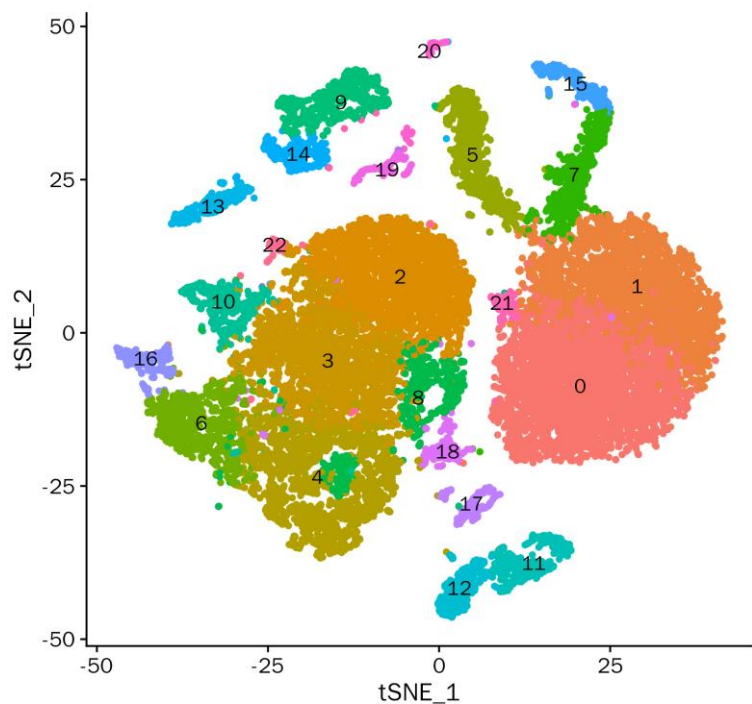

C

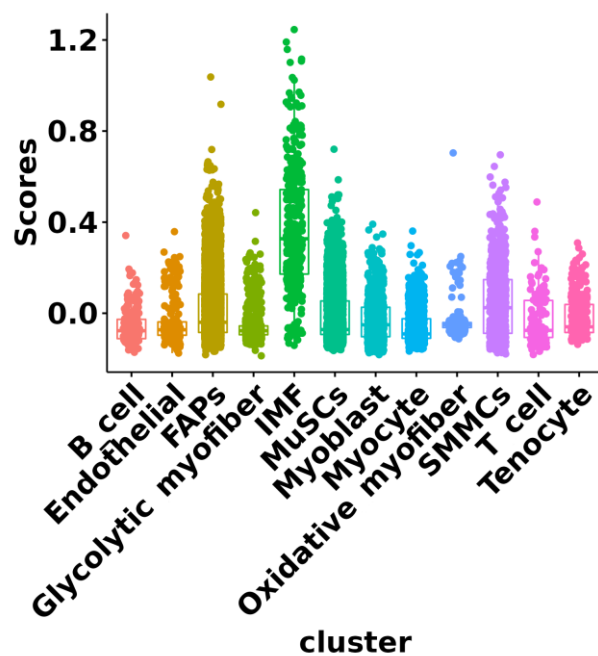

B

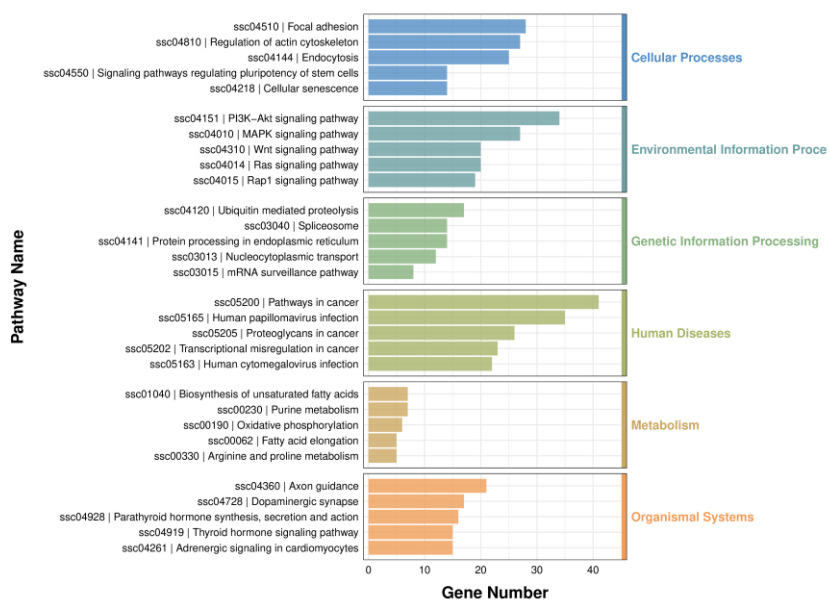

D

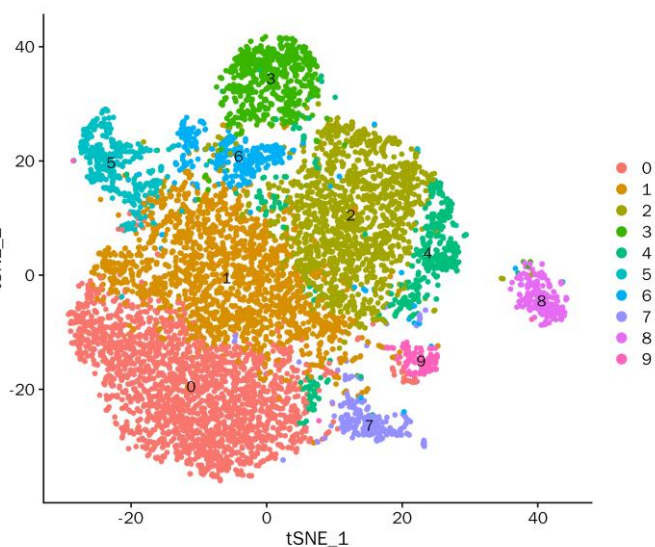

E

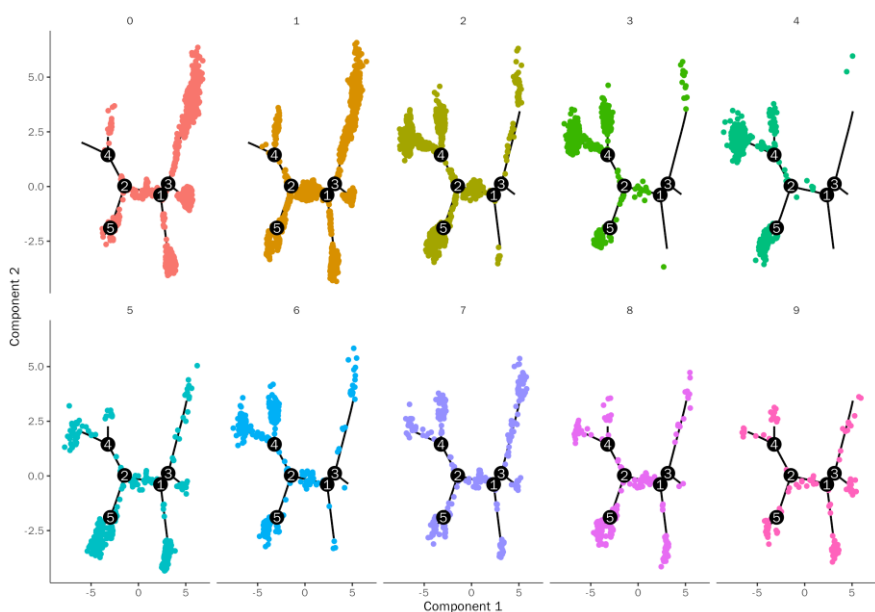

F

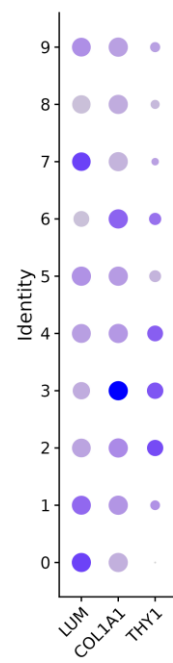

G

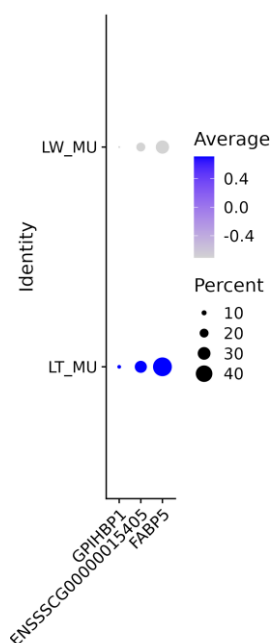

Supplement: Supplementary file 4 — Additional file 3: Fig. S3. The single-cell landscapes and functional annotation of porcine muscle-resident cell types. A tSNE plot showing the distribution of the muscle-resident clusters in LT and LW piglets. B KEGG pathway analysis of IMF-specific genes. C The gene set scores were calculated across various muscle-resident cell types based on the AddModuleScore method. D Graph-based re-clustering of porcine FAPs revealed 10 clusters that were labeled in different colors. E Pseudotime trajectory analysis corresponds to the differentiation of the FAP subpopulation from THY1-positive cells to THY1-negative cells. The cells were colored by cluster types. F Dot plots showing the expression of LUM, COL1A1 and THY1 genes in each cluster of the FAP subpopulation. G The expression levels of CD36 and FABP5 genes in porcine endothelial cells between LT and LW piglets. [file 40168_2023_1743_MOESM3_ESM.pdf]
